# Recent Advances in the Synthesis and Application of Monolayer 2D Metal‐Organic Framework Nanosheets

**DOI:** 10.1002/smsc.202400132

**Published:** 2024-07-10

**Authors:** Yu Wang, Juan Ma, Fei Jin, Tong Li, Negar Javanmardi, Yuyuan He, Guanzhou Zhu, Siwei Zhang, Jian‐Da Xu, Ting Wang, Zhang‐Qi Feng

**Affiliations:** ^1^ School of Chemistry and Chemical Engineering Nanjing University of Science and Technology Nanjing 210094 P. R. China; ^2^ Department of Orthopaedics Changzhou hospital of traditional Chinese Medicine Changzhou hospital affiliated to Nanjing University of Chinese Medicine Changzhou 213003 P. R. China; ^3^ State Key Laboratory of Digital Medical Engineering Southeast University Nanjing 210096 P. R. China

**Keywords:** 2D nanomaterials, Metal–organic frameworks, Monolayer 2D metal‐organic frameworks

## Abstract

Monolayer 2D metal‐organic framework (MOF) nanosheets, characterized by abundant exposed active sites and tunable structure and function (such as altering the metal nodes or organic ligands), have emerged as a pivotal class of 2D materials, demonstrating irreplaceable applications across diverse research domains in materials and chemistry. This review provides a comprehensive survey of the latest research progress in the synthesis of monolayer 2D MOF nanosheets. Specifically, recent synthetic strategies, including top‐down and bottom‐up methods, are delved and their applications in gas separation, catalysis, sensing platforms, and energy storage are explored. Additionally, the challenges faced in the investigation of monolayer 2D MOF nanosheets are elucidated and future opportunities for these materials as a novel generation of 2D materials are outlined.

## Graphical abstract

Monolayer 2D metal‐organic frameworks (MOF) nanosheets have attracted increasing attention due to their ultrathin thickness and high specific surface area. This review summarizes the research progress on monolayer 2D MOF nanosheets in recent years, including synthesis methods and applications. Furthermore, the challenges and opportunities of monolayer 2D MOF nanosheets are discussed in detail.

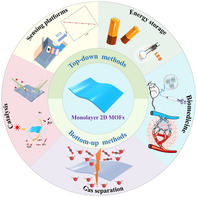

## 1 Introduction

Metal‐organic frameworks (MOFs) represent a class of organic–inorganic hybrid porous materials, composed of inorganic metal ions or clusters and organic ligands interconnected through coordination chemistry principles.^[^ ^1^ ^]^ MOFs exhibit versatility in catalysis,^[^ ^2^, ^3^, ^4^ ^]^ sensing,^[^ ^5^, ^6^, ^7^ ^]^ biomedicine,^[^ ^8^, ^9^, ^10^ ^]^ energy storage,^[^ ^11^, ^12^, ^13^ ^]^ and gas separation and adsorption.^[^ ^14^, ^15^, ^16^ ^]^ This versatility arises from their remarkable attributes such as high porosity, abundant ion diffusion channels, multi‐metal active sites, a high specific surface area, etc. Most MOF nanosheets exist in the form of 3D crystals whose metal active sites cannot be fully utilized, and their applications are limited due to thickness.^[^ ^17^ ^]^ Designing MOF materials into 2D nanosheets can combine the highly ordered structure of MOF itself with the unique physicochemical properties of 2D materials, resulting in higher exposure of metal sites, tunable structure and function, higher flexibility, and processability. This is an effective strategy for obtaining superior performance MOFs.^[^ ^18^, ^19^, ^20^, ^21^, ^22^ ^]^ 2D MOF nanosheets typically comprise single or multiple layers, stacked along one direction. Monolayer 2D MOF nanosheets are 2D MOFs with a single‐atomic‐layer thickness, inherit the structural regularity, and compositional diversity of MOFs. Additionally, they exhibit a high degree of dispersibility and highly accessible active sites, promising benefits for both fundamental research and various applications.^[^ ^23^, ^24^, ^25^ ^]^ While significant efforts have been dedicated to the design of monolayer 2D MOF nanosheets, the field remains in its early stages. The advantages of monolayer 2D MOF nanosheets include: 1) abundance of active metal sites: featuring numerous active metal sites, it provides enhanced catalytic activity; 2) precise control and easy identification: coordination bonds create a highly organized arrangement of atoms, facilitating precise control and easy identification; 3) enhanced gas absorption: its large surface area and porous nature enable the absorption of more gas; and 4) ease of functionalization: after synthesis, it is easily functionalizable, providing new highly dispersed 2D materials. With a comprehensive understanding of the benefits and characteristics of monolayer 2D MOF nanosheets, there is an active research focus on synthesis methods. However, achieving the large‐scale fabrication of monolayer 2D MOF nanosheets with customized characteristics and multiple structures remains a significant challenge.

**Figure 1:**
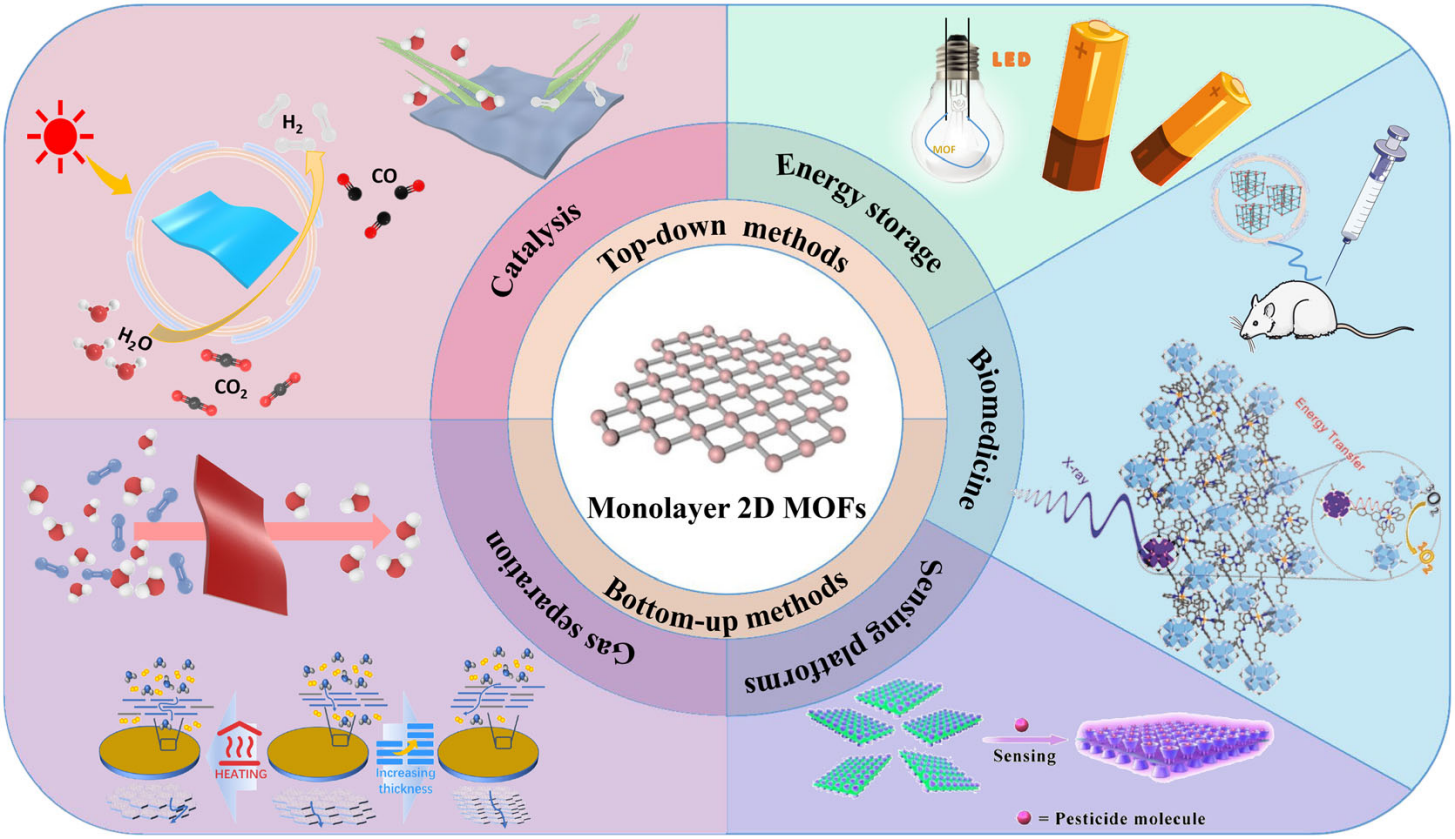
The synthetic strategies and applications of monolayer 2D MOFs. Figures of monolayer 2D MOFs: Reproduced with permission.^[^ ^149^ ^]^ Copyright 2023, Wiley. Figures of gas separation: Reproduced with permission.^[^ ^71^ ^]^ Copyright 2021, Wiley. Figures of sensing: Reproduced with permission.^[^ ^129^ ^]^ Copyright 2020, Elsevier. Figures of biomedicine: Reproduced with permission.^[^ ^142^ ^]^ Copyright 2017, Wiley.

In recent years, research on ultrathin 2D MOFs has experienced rapid growth, leading to a substantial number of critical reviews being published. Following an examination of these published reviews, it is evident that the majority of them focus on discussing the role of 2D MOFs in specific applications individually, such as membrane separation,^[^ ^26^ ^]^ heterogeneous catalysis,^[^ ^27^ ^]^ electro/photocatalysis,^[^ ^28^, ^29^, ^30^ ^]^ electronic devices,^[^ ^31^ ^]^ and integrated circuits.^[^ ^32^ ^]^ Additionally, some reviews offer comprehensive overviews of the synthesis and application of ultrathin 2D MOFs^[^ ^33^, ^34^, ^35^ ^]^ or provide summaries of the unique properties of 2D MOFs and their roles in various applications.^[^ ^36^ ^]^ However, they mainly emphasize the preparation and applications of few‐layer or bulk MOFs, with little mention of monolayer 2D MOFs. Moreover, there are almost no reviews specifically dedicated to monolayer 2D MOFs. Therefore, in order to provide valuable advice for future researchers in this field and related areas, in this comprehensive review, we elucidate the diverse physicochemical attributes inherent in monolayer 2D MOF nanosheets. A thorough exploration of synthetic methodologies for the meticulous preparation of these monolayer 2D MOF nanosheets is presented. Subsequently, we delve into recent breakthroughs and innovations in the application of monolayer 2D MOF nanosheets, providing an in‐depth analysis of their applications (**Figure** **1**). To conclude, we scrutinize potential future challenges and developmental prospects for monolayer 2D MOF nanosheets, positioning them as a new generation of monolayer 2D materials.

## 2 Structural and Functional Characteristics of Monolayer 2D MOFs

Monolayer 2D MOFs usually consist of metal ions (or metal clusters) and organic ligands, which are linked together by strong coordination bonds to form planar surfaces with a regular grids structure. Unlike other 2D nanomaterials such as graphene,^[^ ^37^, ^38^ ^]^ boron nitride,^[^ ^39^, ^40^ ^]^ and transition metal dichalcogenides,^[^ ^41^, ^42^ ^]^ characterized by compositional simplicity, monolayer 2D MOFs offer a unique advantage: their tunable structure and function. They can be designed and adjusted based on different metal ions and organic ligands, and by altering the connection methods (such as direct connections or through bridging groups), different topological structures can be achieved, demonstrating a high degree of structural customizability.

As pivotal nodes in the MOF, the choice of metal ions or clusters and their coordination environment significantly influences the electronic properties of MOFs, thereby impacting their applications in catalysis and energy storage. Notably, transition metals like titanium and chromium, when utilized as nodes, provide vacant d‐orbitals, enhancing the catalytic activity of MOFs.^[^ ^43^ ^]^ Hexaaminobenzene (HAB)‐derived 2D MOFs exhibit outstanding performance in advanced energy storage systems. Mortazavi et al.^[^ ^44^ ^]^ investigated the mechanical and electronic properties of various transition metal‐HAB nanosheets, revealing distinct properties based on the metal nodes. Monolayer Ag–, Cu–, Cr–, and Mn‐HAB demonstrate perfect half‐metallic behaviors, while Ni–, Pd–, and Rh‐HAB monolayers exhibit nonmagnetic metallic behavior. Moreover, the chemical affinity, electrical characteristics, and optical features of the MOF can be tailored by incorporating diverse functional groups. For example, the addition of dipolar functional groups enhances the framework's selective adsorption capabilities for dipolar molecules.^[^ ^45^ ^]^ Additionally, monolayer 2D MOFs also possess an ultrathin thickness and enhanced specific surface area and porosity, providing unique physical structural foundations for the applications of monolayer 2D MOFs. Specifically:

### 2.1 Ultrathin Thickness of Monolayer 2D MOFs

Monolayer 2D MOF nanosheets exhibit ultrathin monolayer structure, boasting a minimal thickness as low as 0.7 nm.^[^ ^46^ ^]^ This ultrathin structure allows for swift molecular or ionic permeation throughout the entire material. In contrast, bulk MOFs possess larger dimensions and thicker profiles, typically ranging from a few nanometers to tens of nanometers. The ultrathin nature of monolayer 2D MOF nanosheets ensures the complete exposure of metal active sites, a feature divergent from the internal channels within bulk MOFs. This characteristic facilitates the chemical modification of the catalytic center's surroundings, leading to a substantial reduction in the electron diffusion length. Consequently, the catalyst's utilization of photogenerated electrons is markedly enhanced.^[^ ^47^ ^]^ For example, monolayer Zr‐RuBPY MOFs incorporating Ru(bpy)_3_^2+^ moieties demonstrate high photocatalytic efficacy in reactions involving aryl diazonium salts, bis‐enone cycloaddition between styrene and nitrile, and Meerwein addition under visible light irradiation.^[^ ^48^ ^]^ Simultaneously, the ultrathin monolayer structure imparts a more controllable pore architecture to monolayer 2D MOF, thereby enhancing its adsorption capacity and catalytic activity. Moreover, monolayer MOF nanosheets can be assembled with other functional electronic materials through surface anchoring sites. They can also be directly interfaced with conductive electrodes, fostering direct contact between the electrode surface and the active sites, thus facilitating efficient charge transmission. Notably, monolayer 2D MOF nanosheets exhibit exceptional performance in electrocatalytic processes such as the oxygen evolution reaction (OER), hydrogen evolution reaction (HER), and oxygen reduction reaction (ORR).^[^ ^49^ ^]^

### 2.2 Enhanced Specific Surface Area and Porosity in Monolayer 2D MOFs

Due to the porous nature of monolayer 2D MOFs, molecules can enter the material through channels and voids, giving it not only a high surface area but also allowing for the formation of more pores and spaces inside, further increasing its surface area. Currently, the lateral size of monolayer 2D MOFs can reach several micrometers,^[^ ^50^, ^51^ ^]^ with a specific surface area of around 1173 m^2^g^−1^.^[^ ^52^ ^]^ Additionally, due to its planar structure and porous nature, monolayer 2D MOFs typically have a high porosity. These channels and voids provide abundant storage space and reaction sites, allowing MOFs to adsorb, store, and transport various molecules, achieving higher adsorption capacity and diffusion rates. The high specific surface area and porosity of monolayer 2D MOF nanosheets give them excellent permeability, making them highly attractive for energy storage, gas separation, and adsorption applications by exposing them to surrounding solutions.^[^ ^53^, ^54^ ^]^ For instance, monolayer Zn‐MOF nanosheets, with lateral dimensions reaching up to 0.35 nm, achieved a maximum uptake capacity of 6639.55 mg g^−1^. It can remove more than 99.35% of Congo Red (CR) in the concentration range from 40 to 400 ppm.^[^ ^55^ ^]^

These studies indicate that monolayer 2D MOFs show promising applications in gas separation, energy storage, catalysis, and sensing fields.

## 3 Synthesis Methods of Monolayer 2D MOFs

**Figure 2:**
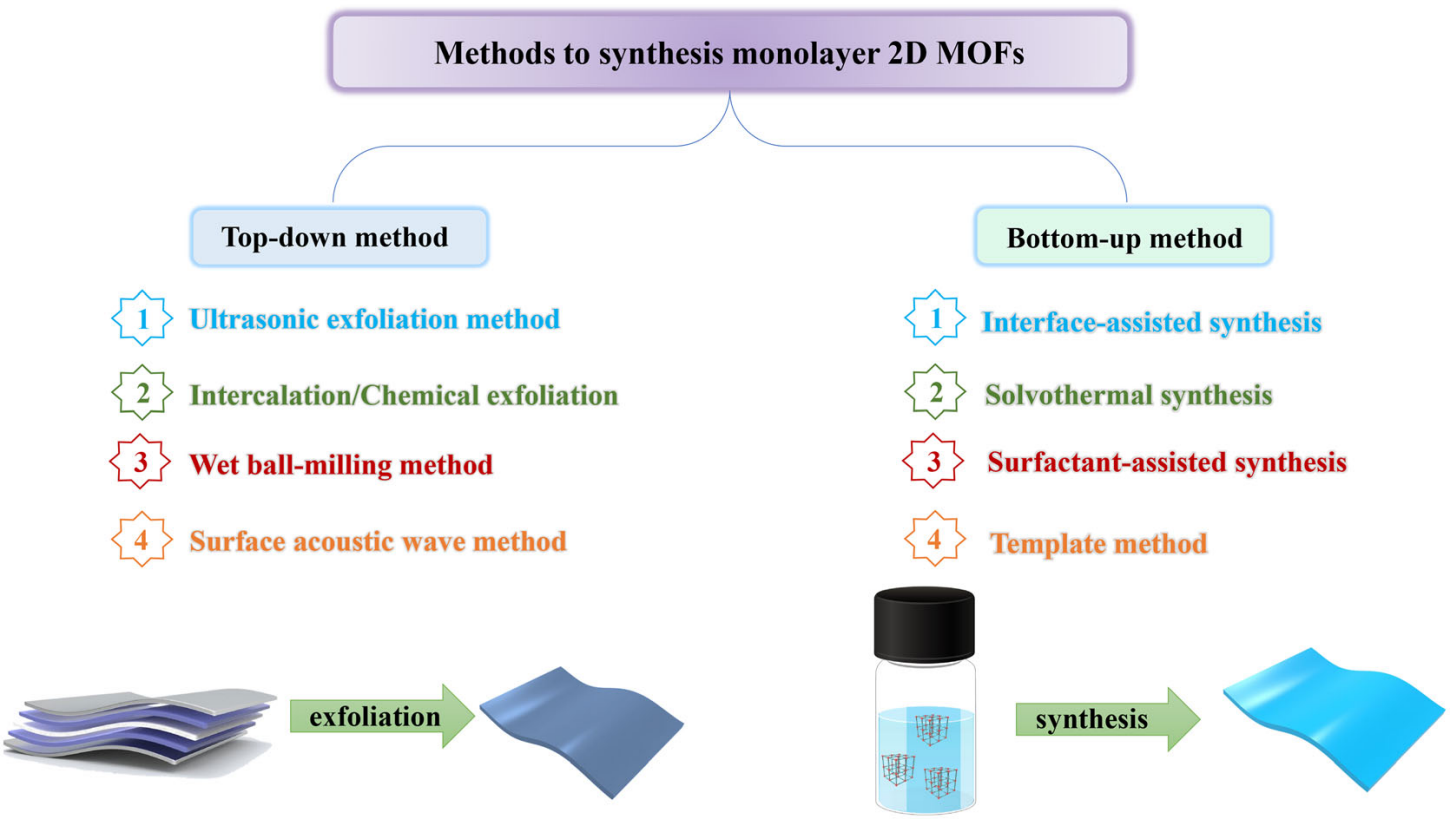
Method of the different routes used to prepare monolayer 2D MOF nanolayers.

In order to prepare monolayer 2D MOFs, an array of synthetic strategies has emerged, primarily categorized as top‐down and bottom‐up methods (**Figure** **2**). This section delves into the latest advancements in synthesizing monolayer 2D MOFs, providing a comprehensive discussion and summarization of the most recent synthesis strategies.

### 3.1 Top‐Down Methods

Top‐down methods stand as a pivotal approach for the conversion of inherently layered MOFs bulk materials into monolayer 2D MOF nanosheets, accomplished through physical or chemical interventions. MOFs exhibit a layered structural configuration characterized by robust coordination bonds within the layers and comparatively weaker van der Waals forces and hydrogen bonds between them. The top‐down method can overcome the interlayer forces to obtain monolayer MOF crystals.^[^ ^56^ ^]^ Nevertheless, the intralayer coordination bonds in layered 2D MOF nanosheets are not always stronger than the interlayer interactions, making some 2D MOF nanosheets difficult to exfoliate. The main methods include ultrasonic exfoliation, intercalation/chemical exfoliation, wet ball‐milling method, and surface acoustic wave (SAW) method. Each of these approaches offers distinctive advantages and considerations in the quest for monolayer MOF nanosheet production.

#### 3.1.1 Ultrasonic Exfoliation Method

**Figure 3:**
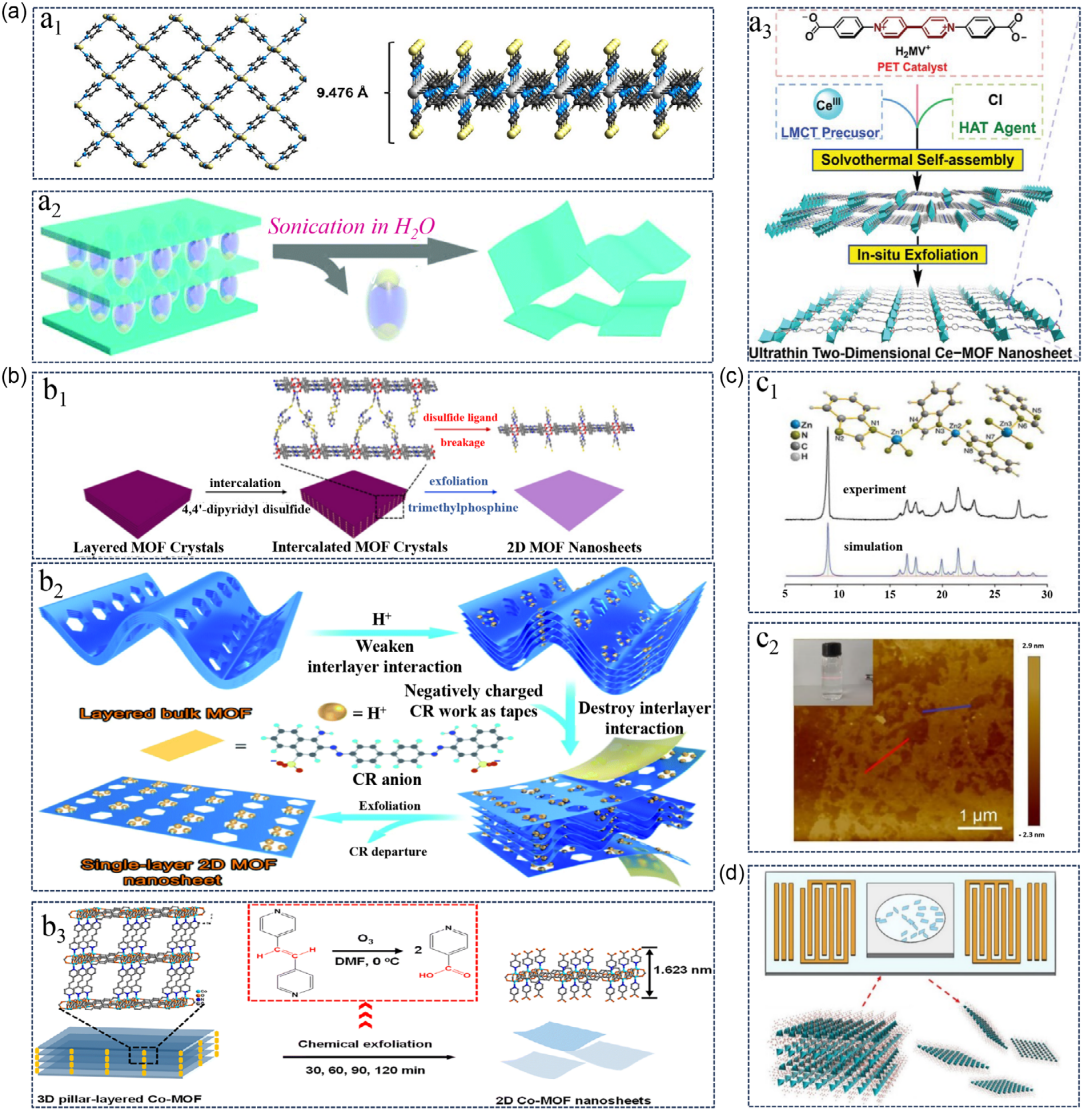
Top‐down methods for synthesizing monolayer 2D MOFs. a) Ultrasonic exfoliation method for monolayer MOF preparation. a_1_: Connectivity scheme of 2D monolayer structure from various perspectives. Reproduced with permission.^[^ ^58^ ^]^ Copyright 2018, ACS. a_2_: Schematic illustration of monolayer 2D MOF nanosheet production via the synergistic interaction of host–guest interactions and ultrasonic treatment. Reproduced with permission.^[^ ^61^ ^]^ Copyright 2020, RSC. a_3_: Schematic representation of monolayer 2D MOF nanosheet production using ultrasonic stripping method from layered bulk Ce‐MV^+^. Reproduced with permission.^[^ ^62^ ^]^ Copyright 2023, Wiley. b) Intercalation/Chemical exfoliation method. b_1_: Schematic diagram of monolayer 2D nanosheet preparation through the intercalation/chemical exfoliation method. Reproduced with permission.^[^ ^65^ ^]^ Copyright 2017, ACS. b_2_: Schematic overview of monolayer Zn‐MOF nanosheet preparation. Reproduced with permission.^[^ ^55^ ^]^ Copyright 2020, RSC. b_3_: Schematic depiction of 3D columnar layer Co‐MOF exfoliation. Reproduced with permission.^[^ ^45^ ^]^ Copyright 2023, RSC. c) Soft chemical exfoliation method. c_1_: X‐ray diffraction pattern of monolayer Zn_2_(bim)_4_ nanosheets prepared by chemical exfoliation. Reproduced with permission.^[^ ^70^ ^]^ Copyright 2014, AAAS. c_2_: AFM image of monolayer Zr‐BTB MOF nanosheets prepared by chemical exfoliation. Reproduced with permission.^[^ ^71^ ^]^ Copyright 2021, Wiley. d) SAW method: Schematic of SAW exfoliation device and Zn_2_(bim)_4_. Reproduced with permission.^[^ ^74^ ^]^ Copyright 2022, Elsevier.

The ultrasonic exfoliation method harnesses the external force of ultrasonic waves to delicately peel layered bulk MOF materials. Precise control over the exfoliation process is achieved by adjusting the frequency and duration of ultrasonic waves.^[^ ^57^ ^]^ This method typically involves three sequential steps: solvent selection, optimization of ultrasonic parameters, and centrifugal deposition. Notably, ultrasonic exfoliation offers several advantages, including high efficiency, controllability, and the absence of harmful reagents. An illustrative example by Luo et al. demonstrates the preparation of monolayer 2D [Co (CNS)_2_(pyz)_2_]*n* (pyz = pyrazine) nanosheets using the ultrasonic exfoliation method.^[^ ^58^ ^]^ Using ultrasonic force‐assisted liquid exfoliation in an ethanol solution, they achieved monolayer 2D MOFs with a thickness below 1.0 nm from bulk precursors (**Figure** **3**a_1_). The ultrathin atomic thickness and the abundance of sulfur atoms with high electronegativity render these monolayer 2D MOF nanosheets highly sensitive to intermolecular interactions. Postsynthetic functionalization offers a highly appealing approach for tuning the structure and properties of 2D MOFs. Foster et al.^[^ ^59^ ^]^ conducted pioneering research on the impact of postsynthetic functionalization on the exfoliation of 2D MOF nanosheets. This functionalization formed stable covalent bonds and introduced repulsive charges between layers, effectively achieving the exfoliation of monolayer 2D MOF nanosheets with a thickness of 1.4 nm. This method has broad applicability for improving the exfoliation of layered MOFs and can introduce functional groups to create acid–base active sites for catalysis. Moreover, weak interlayer interactions play a crucial role in the exfoliation of monolayer 2D MOF nanosheets. By designing 2D cationic MOFs with spatial cage structures, interlayer interactions can be minimized. The spatial cage structure imparts the MOF with extremely weak interlayer interactions (≈1/46th of graphene), facilitating large‐scale exfoliation under ultrasonication to yield uniformly thick monolayer 2D MOF nanosheets.^[^ ^60^ ^]^ However, challenges arise due to the instability of intralayer coordination bonds during exfoliation, posing difficulties in the preparation of monolayer MOFs. An effective method to address this challenge is to utilize the anisotropy of coordination bonds in 3D pillar‐layered MOFs to selectively break interlayer coordination bonds while preserving the integrity of intralayer coordination bonds. By exchanging the dabco pillars in 3D pillar‐layered MOFs using H_2_O as a top guest molecule, intralayer coordination bonds remain intact, preventing the reassembly of resulting 2D layer‐like nanosheets (Figure 3a_2_). This process yields monolayer nanosheets with a thickness of ≈0.9 nm, achieving a high yield of 94%.^[^ ^61^ ^]^ Additionally, the disruption of weak electrostatic interactions between layers in bulk MOFs using ultrasound facilitates the production of high‐purity monolayer MOFs. Ji et al. successfully employed this technique to exfoliate bulk Ce‐MV^+^ MOFs, constructed with methyl viologen (MV^+^) as the organic ligand, into monolayer 2D Ce‐MV^+^ nanosheets.^[^ ^62^ ^]^ This innovative strategy disrupts weak interlayer electrostatic interactions, enabling easy ultrasound‐assisted exfoliation and resulting in monolayer nanosheets with a thickness of 0.7 nm (Figure 3a_3_). Ultrasonic exfoliation proves to be a rapid and efficient method for peeling multilayer MOF materials into monolayer nanosheets, significantly enhancing preparation efficiency. Notably, this method allows for the preservation of the morphology and crystal structure of MOF materials, minimizing the risk of material damage or deformation during the exfoliation process. However, it is essential to consider the potential impact on the stability of MOF materials, especially delicate MOF structures, which may experience damage during the exfoliation process.

#### 3.1.2 Intercalation/Chemical Exfoliation Method

The 2D MOF nanosheets generally obtained by exfoliation can overcome the strong mechanical force required for interlayer interaction, which would lead to structural deterioration. At the same time, due to their ultrathin thickness and strong interlayer interactions, the prepared 2D MOF nanosheets are prone to stacking.^[^ ^63^ ^]^ Addressing this challenge involves the strategic use of intercalation agents and controlled chemical reactions to modulate interlayer interactions, resulting in the transformation of 3D MOFs into 2D monolayer MOFs.^[^ ^64^ ^]^ Pioneering work by Ding et al.^[^ ^65^ ^]^ introduced a method for chemically assisted exfoliation of monolayer 2D MOF nanosheets. This innovative approach involves the incorporation of disulfides into layered porphyrin MOFs, resulting in the creation of a new intercalated MOF (Figure 3b_1_). Subsequent selective cleavage of the disulfide bonds induces the detachment of the embedded MOF, yielding monolayer MOF nanosheets with a thickness of ≈1 nm. This technique boasts a commendable yield rate of 57%, offering a promising avenue for the large‐scale production of monolayer MOF nanosheets. Typically, MOF exfoliation strategies concentrate on attenuating van der Waals forces or hydrogen bond interactions between layers in bulk crystals. For instance, the reduction of interlayer van der Waals forces in a Zn‐MOF can be achieved through protonation in an acidic environment. Subsequently, CR molecules with a negative charge can electrostatically adhere to the surface of the protonated MOF, further disrupting weakened interlayer van der Waals interactions. The outcome is the formation of ultrathin monolayer 2D Zn‐MOF nanosheets with a maximum lateral size of 3.4 nm (Figure 3b_2_).^[^ ^55^ ^]^ Remarkably, these nanosheets exhibit exceptional adsorption capabilities, swiftly and selectively removing CR molecules with an absorption rate of up to 6639.55 mg g^−1^. The potential of MOFs in eliminating contaminants is substantial. Despite notable strides in chemically exfoliating 3D MOFs, the development of a straightforward and universally applicable exfoliation method remains a formidable challenge. In a study by Zhuo et al.^[^ ^45^ ^]^ a comprehensive exfoliation approach was introduced for generating monolayer MOFs from the 3D pillar‐layered Zn/Cu/Co‐MOF, utilizing bipyen as the pillar ligand (Figure 3b_3_). The process involved the oxidation reaction of ozone to break the C≡C bonds in Zn/Cu/Co‐MOFs with trans‐1,2‐bis(4‐pyridyl) ethylene (bipyen) as a pillar ligand. This resulted in the exfoliation of monolayer 2D MOFs functionalized with a –COOH group from the 3D columnar layer MOFs. Precise control over the exfoliation process and MOF thickness was achieved by adjusting the oxidation time of ozone. Apart from ozone, the high diffusion rate, zero interfacial tension, and low viscosity of supercritical carbon dioxide have been proven to be an efficient microenvironmental system for the intercalation and exfoliation of layered materials.^[^ ^66^ ^]^ Chang et al.^[^ ^67^ ^]^ first prepared large‐sized and 1.1 nm‐thick monolayer 2D Ni‐BDC MOF nanosheets in an aqueous system with the assistance of SC CO_2_. A thin layer of SC CO_2_ accumulating at the water–Ni–BDC interfaces can convert the original hydrophilic interface into a superhydrophobic one. This superhydrophobic layer formed at the water–MOF interface effectively prevents dissociation, thereby enhancing the stability of Ni‐BDC in aqueous systems. The intercalation/chemical exfoliation method used proved to be simple and feasible, involving the cleavage of covalent bonds in pillar ligands through solvent treatment, oxidation, or chemical reactions for the exfoliation of multilayer MOFs. Despite its efficacy, this method encounters several challenges. The preparation process may alter or damage the crystal structure, potentially impairing its functionality. For intricate MOF structures like organic‐functionalized or multimetal MOFs, further exploration of exfoliation methods is warranted. With continued in‐depth research and technological advancements, chemical exfoliation methods hold the promise of unlocking additional avenues for the preparation of monolayer MOFs.

#### 3.1.3 Wet Ball‐Milling Method

In traditional physical exfoliation processes, shear stress induced by external forces can compromise the integrity of MOF nanosheets, resulting in the cleavage of coordination bonds and disruption of their in‐plane structure.^[^ ^68^, ^69^ ^]^ This challenge is effectively addressed through the utilization of the soft physical exfoliation method, which synergistically uses wet ball milling in conjunction with ultrasonic exfoliation. Wet ball milling introduces larger surface contact and uniform forces, prompting MOF nanosheets to exfoliate under the influence of the milling medium. Simultaneously, ultrasonic exfoliation harnesses high‐frequency forces generated by ultrasonic vibrations to augment the exfoliation effect. The amalgamation of these techniques enhances the mechanical force on MOF nanosheets while minimizing structural damage. For instance, Peng et al.^[^ ^70^ ^]^ employed methanol and propanol as volatile solvents for soft physical exfoliation, yielding monolayer 2D Zn_2_(bim)_4_ nanosheets. During wet ball milling, the original Zn_2_(bim)_4_ crystals are milled at a low speed (60 rpm), facilitating the penetration of small methanol molecules into the galleries of the layered Zn_2_(bim)_4_. Subsequently, exfoliation in a volatile solvent, assisted by ultrasonic waves, produces monolayer 2D Zn_2_(bim)_4_ nanosheets with a thickness of 1 nm and a pore size of ≈0.21 nm (Figure 3c_1_). These Nano sheets exhibit high thermal stability, maintaining structural integrity at temperatures as high as 200 °C. Methanol, chosen for its polar nature and excellent solubility, promotes dispersion and exfoliation during wet ball milling without compromising the nanosheets’ subsequent applications. Additionally, other polar solvents can serve as effective milling solvents. For example, monolayer 2D Zr‐BTB MOF nanosheets with a 1 nm thickness can be produced using water molecules (Figure 3c_2_).^[^ ^71^ ^]^ The soft physical exfoliation method, characterized by its simplicity and controllability, successfully mitigates potential internal structure damage caused by conventional physical exfoliation methods. It preserves the structural integrity of MOF nanosheets, thereby enhancing their application performance. However, due to technical limitations during the process of soft physical exfoliation for monolayer MOF preparation, it often results in low yields. This implies a potential waste of significant amounts of feedstock materials and restricts the large‐scale synthesis of monolayer MOFs.

#### 3.1.4 Surface Acoustic Wave Method

SAWs are electromechanical waves with nanometer or subnanometer amplitudes that propagate along the surface of a piezoelectric substrate. They can be used in conjunction with strong electric fields in liquids to drive fluid motion at the microscale.^[^ ^72^ ^]^ Noteworthy advantages of SAWs over traditional ultrasonic waves include streamlined operation, low power consumption, and enhanced control over the thickness of exfoliated 2D nanosheets.^[^ ^73^ ^]^ For instance, the application of SAW technology facilitates the controlled exfoliation of layered Zn_2_(bim)_4_‐MOF nanosheets into monolayer 2D MOF nanosheets within a microfluidic system (Figure 3d). The synergy of electric and acoustic fields generated by SAWs in liquid weakens interlayer bonding, leading to the generation of robust shear forces that effectively separate the MOFs.^[^ ^74^ ^]^ Demonstrably, the thickness of MOF nanosheets can be precisely manipulated by adjusting the duration of SAWs, offering a scalable approach to the preparation of monolayer 2D MOF nanosheets. The commendable attributes of SAW technology encompass superior control over monolayer nanosheets, operational simplicity, a noncontact nature, and favorable scalability. However, it is imperative to acknowledge certain limitations, including the requisite for specialized experimental equipment and the intricate growth mechanism of MOFs. Future research endeavors should focus on refining the operational and control strategies of the SAW method, aiming to amplify its potential applications in the preparation of monolayer MOFs.

### 3.2 Bottom‐Up Methods

Nonlayered MOF nanosheets are difficult to obtain through top‐down methods. However, a more viable alternative lies in employing bottom‐up methods, wherein the pivotal factor is the selective regulation of the growth direction of MOF nanosheets. Direct synthesis of monolayer 2D MOF nanosheets from metal and organic precursors offers a means to precisely govern the morphology, structure, and properties of the monolayer MOF. Nevertheless, bottom‐up methods often suffer from low yields and pose challenges in removing surface‐active agent molecules or end‐capping ligands from the nanosheet surface. Moreover, they may necessitate intricate synthesis conditions and procedures, imposing stringent requirements on the selection and handling of precursors. Bottom‐up methods including the interface‐assisted synthesis method, solvothermal synthesis method, surfactant‐assisted synthesis, and template method, each contribute unique advantages to the synthesis process.

#### 3.2.1 Interface‐Assisted Synthesis Method

##### Liquid–Gas Interface Synthesis Method

**Figure 4:**
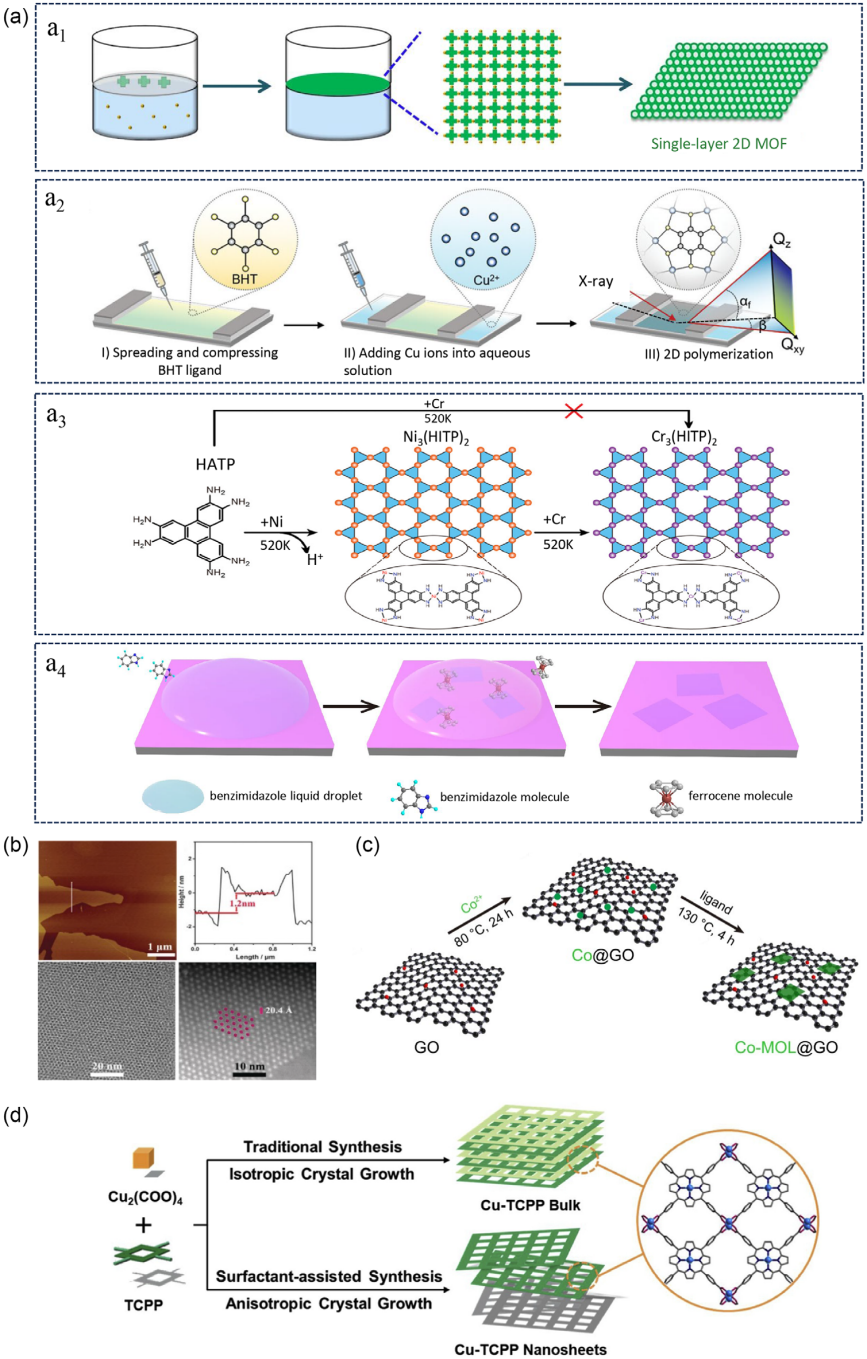
Bottom‐up methods for synthesizing monolayer 2D MOFs. A) Interface‐assisted synthesis for preparation of monolayer MOF: a_1_: Schematic diagram of air–water interface synthesis for monolayer MOF. Reproduced with permission.^[^ ^77^ ^]^ Copyright 2023, ACS. a_2_: Schematic diagram of L‐B synthesis for preparation of monolayer MOF. Reproduced with permission.^[^ ^84^ ^]^ Copyright 2024, Wiley. a_3_: Synthesis of Cr_3_(HITP)_2_ framework through surface substitution reaction. Reproduced with permission.^[^ ^91^ ^]^ Copyright 2023, Wiley. a_4_: SCA‐CVD growth of atomically 2D MOF single crystals. Reproduced with permission.^[^ ^94^ ^]^ Copyright 2024, Nature. b) Solvothermal synthesis for preparation of monolayer MOF schematic. Reproduced with permission.^[^ ^98^ ^]^ Copyright 2016, GDCh. c) Preparation of Co‐MOF using graphene oxide as a template. Reproduced with permission.^[^ ^100^ ^]^ Copyright 2021, Nature. d) Schematic diagram of surfactant‐assisted methods for preparation of Cu‐TCPP MOFs. Reproduced with permission.^[^ ^105^ ^]^ Copyright 2022, ACS.

Due to the relatively large thickness of monolayer MOFs synthesized at the liquid–liquid interface and the tendency for defects in nanosheets synthesized at the liquid–solid interface, the liquid–gas interface is commonly used in preparing ideal monolayer 2D MOF nanosheets.^[^ ^75^ ^]^ The liquid–gas interface is a flat space between gas and water that can provide 2D confinement for monomers.^[^ ^76^ ^]^ In the synthesis of monolayer 2D MOFs at the liquid–gas interface, the phenomenon of “interfacial crystallization” is typically employed. This phenomenon occurs at the liquid surface, where a specific interface is formed when suitable ligands and metal ions are present on the surface, allowing MOFs to grow only within a limited 2D interface, forming monolayer 2D nanosheets (**Figure** **4**a_1_). This method can produce monolayer MOFs with large lateral dimensions, and the chemical properties and structure can be adjusted by designing ligands and controlling synthetic conditions such as solvent concentration.^[^ ^77^ ^]^ For example, Kitagawa et al.^[^ ^78^ ^]^ synthesized monolayer 2D MOFs at the liquid–gas interface using CoTCPP (TCPP = 5,10,15,20‐tetrakis(4‐carboxyphenyl) porphyrinato) as the carboxylic acid ligand and pyridine as the auxiliary ligand molecule. They diffused a mixed solution of CoTCPP and pyridine molecular building units onto a Cu^2+^ ion water solution at the liquid–gas interface. The copper ions bound to the CoTCPP ligands, forming well‐crystallized 2D monolayer CoTCPP‐py‐Cu nanosheets at the water/air interface. The thickness of the layer‐by‐layer 2D MOF thin layer materials can be controlled through the repeated process of continuous thin‐film deposition. UiO‐66 is a chemically stable MOF that can be easily prepared in various forms and sizes, Lu et al.^[^ ^79^ ^]^ used a liquid–gas interface self‐assembly process to prepare monolayer UiO‐66, which was used as a template for metal electrodeposition. Liquid–gas interface synthesis can control the positioning and arrangement of monolayer MOFs within a 2D confined space, achieving highly ordered structures. However, the development of interface technology is significantly influenced by the characteristics of the precursors, such as sensitivity, solubility, catalytic activity, functional groups, thermal stability, and their specific interactions with the interface, making the operation complex and less feasible.^[^ ^80^ ^]^

##### Langmuir–Blodgett Synthesis Method

The Langmuir–Blodgett (L–B) synthesis method is a technique used to construct organic monolayers with ordered structures. This process involves the diffusion of materials from a volatile, water‐immiscible solvent onto the water surface, thereby creating a floating monolayer that is subsequently transferred onto a solid substrate.^[^ ^81^ ^]^ The L–B technique can control the packing density of monomers by adjusting the surface pressure of water, thereby manufacturing nanosheets with a monolayer thickness.^[^ ^80^ ^]^ The process primarily involves the following steps: 1) dispersing monolayers of 2D material monomers on the liquid surface in the L–B trough; 2) using compression techniques to control the surface pressure and stack the monomers into dense and ordered monolayer structures; and 3) injecting a solution of metal ions, which coordinate with ligands to form a monolayer MOF.^[^ ^82^ ^]^ Dong et al.^[^ ^25^ ^]^ demonstrated a typical work of synthesizing large‐area, freestanding monolayer 2D MOF nanosheets using the L–B synthesis method. 1,2,5,6,9,10‐triphenylhexathiephene (THT) was used as a crucial π‐conjugated monomer for constructing key units. The THT monomers were spread as submonolayers on the water surface in the L–B trough, tightly packed into a dense film, compressed to 10 mN m^−1^. Nickel salt solution was injected into the aqueous phase, and as nickel ions diffused from the bulk to the interface, coordination of nickel ions with the dithiolene units triggered 2D supramolecular polymerization, resulting in the formation of large‐area monolayer 2D MOFs. The nanosheets have lateral dimensions of approximately square millimeters, thickness ranging from 0.7 to 0.9 nm, and exhibit a unique freestanding structure. It is worth mentioning that these nanosheets can be fully transferred to any substrate, such as a glassy carbon electrode, for electrochemical studies. The researcher then utilized the same process to prepare a monolayer THTA‐Ni 2D MOF composed of NiS_2_N_2_ complexes and a THT‐Co 2D MOF composed of CoS_4_ complexes. After transferring the obtained THTA‐Co 2D MOF nanosheets vertically from the water surface, an independent monolayer with uniform distribution of cobalt, sulfur, nitrogen, and carbon elements was obtained, with a thickness of 0.8 ± 0.1 nm as detected by atomic force microscopy.^[^ ^82^ ^]^ Furthermore, monolayer 2D c‐MOFs can be combined with graphene to prepare novel organic–inorganic bilayer 2D van der Waals heterostructures. Incorporating the chemical tunability of monolayer 2D MOFs into such van der Waals heterostructures will lead to properties and functionalities designed at the molecular level, further modulated through interlayer interactions.^[^ ^83^ ^]^ Wang et al. synthesized a monolayer Cu_3_BHT (BHT = benzenehexathiol) with strong π‐d conjugation and a 2D planar structure using the L–B method. Atomic force microscopy detected a thickness of ≈0.8 nm, with good micrometer‐scale uniformity (Figure 4a_2_).^[^ ^84^ ^]^ The L–B synthesis method allows the deposition of monolayer MOFs onto specific substrates through vertical lifting movements. Using an isolation board to transfer the monolayer film from the water surface to the solid substrate surface, precise control over the structure and morphology of the monolayer MOFs can be achieved at the molecular or atomic level. This is done by adjusting factors such as the type of solvent, temperature, and pressure within the reaction system, which allows for the manipulation of the MOF's crystal structure and pore size. However, due to the low mechanical strength of the monolayer nanosheets, cracks or defects are prone to occur during the transfer process. Additionally, the varying hydrophilicity and hydrophobicity of different substrate surfaces can affect the transfer and adhesion efficiency of the MOFs.

##### Surface Synthesis Method

The surface synthesis method involves the precise deposition of chemical functional groups and metal atom‐functionalized linker agent molecules onto an atomic plane within ultrahigh‐vacuum conditions. Following a carefully controlled temperature annealing process, a monolayer MOF nanosheet is formed on the surface.^[^ ^43^ ^]^ Presently, this methodology is primarily employed for the synthesis of monolayer 2D conjugated MOF nanosheets. Monolayer 2D conjugated MOFs refer to layer‐stacked MOFs comprising ortho‐substituted conjugated building blocks (such as benzene, triphenylene) and square‐planar bonds with high in‐plane conjugation and weak out‐of‐plane van der Waals interactions. These conjugated components establish a controllable structure within the layer, displaying unique electronic conductivity properties.^[^ ^85^ ^]^ Lyu et al.^[^ ^86^ ^]^ utilized the surface synthesis method to fabricate monolayer 2D conjugated MOFs of M_3_(HAT)_2_ (M = Ni, Fe, Co) on Ag and Au substrates. The chosen building unit, 1,4,5,8,9,12‐hexaazatriphenylene (HAT), possesses a small π‐conjugated core, strong bidentate chelation, and coordination capabilities, rendering it ideal for constructing 2D conjugated MOFs. Currently, the synthesized monolayer 2D conjugated MOFs predominantly rely on late‐transition metals like iron (Fe), cobalt (Co), and nickel (Ni).^[^ ^87^, ^88^ ^]^ However, early‐transition metals such as titanium (Ti), chromium (Cr), and manganese (Mn) exhibit heightened catalytic activity, stronger redox capability, and enhanced coordination ability. Therefore, the integration of early‐transition metals into monolayer 2D conjugated MOFs holds paramount importance in enhancing their performance.^[^ ^89^, ^90^ ^]^ For instance, on an Au substrate, a framework of Ni_3_(HITP)_2_ (HITP = 2,3,6,7,10,11‐hexaaminotriphenylene) was synthesized, and subsequently, Cr atoms were deposited onto the Ni_3_(HITP)_2_ framework, replacing Ni atoms (Figure 4a_3_). Under ultrahigh‐vacuum conditions, a monolayer Cr_3_(HITP)_2_ was synthesized, showcasing an innovative strategy for the fabrication of early‐transition metal‐based monolayer 2D‐conjugated MOFs.^[^ ^91^ ^]^

Chemical vapor deposition (CVD) is a typical surface synthesis method where precursor gases are heated, causing them to thermally decompose into metal atoms and organic ligand groups that react on the surface to form monolayer MOFs with precisely controlled thickness.^[^ ^92^ ^]^ Dong et al.^[^ ^93^ ^]^ successfully synthesized a novel type of 2D c‐MOF on a liquid gallium surface using CVD technology. They prepared nine types of ultrasmooth 2D c‐MOFs with extremely low roughness values (≈2 Å) on the liquid gallium surface. This planar structure can be reproduced in structure with thicknesses ranging from 1.7 to 208 nm, highlighting the method's high versatility and reliability. Compared to traditionally synthesized c‐MOFs, the surface flatness of these nanosheets improved tenfold. The production of high‐quality, large‐size monolayer MOFs via CVD is significant, and self‐condensation‐assisted CVD (SCA‐CVD) enables the growth of large‐size monolayer MOFs with a high monolayer ratio through a simple one‐step process. The grown 2D MOF single crystals have clean and good crystallinity. In the CVD process, the self‐condensation of the precursor, induced by a designed temperature gradient, is considered a key mechanism promoting the growth of atomically thin, large‐size MOF single crystals. Luo et al.^[^ ^94^ ^]^ utilized self‐condensation‐assisted CVD to grow high‐quality MOF single crystals with atomic thickness, where large‐area isolated monolayer single crystals of Fe_*n*_(bim)_2*n*_ achieved high crystallinity with grain sizes up to 62 μm (Figure 4a_4_). Surface synthesis methods offer strong monolayer control, good substrate activity, and high scalability. Conductive MOFs are emerging photoelectronic electroactive materials, and the fabrication of monolayer 2D c‐MOFs by surface synthesis can greatly promote the integration of MOFs in microelectronics. However, there are some limitations, such as the high substrate preparation requirements due to the involvement of high temperatures, relatively complex operations, and high costs.

#### 3.2.2 Solvothermal Synthesis Method

Solvothermal synthesis stands as a pivotal technique in the fabrication of single crystals, achieved by dissolving minerals in solvents under elevated temperature and pressure conditions.^[^ ^95^, ^96^ ^]^ The nuanced control over temperature and reaction duration facilitates the precision engineering of MOFs, controlling their morphology, crystal structure, and porosity with precision.^[^ ^97^ ^]^ The solvent milieu ensures favorable equilibrium and diffusion processes, propelling crystal growth and formation. Consequently, MOFs synthesized through solvothermal methods exhibit distinguished traits, including elevated thermal stability, crystalline integrity, and notable porosity. In a study conducted by Cao et al.^[^ ^98^ ^]^ solvothermal synthesis was instrumental in crafting monolayer 2D MOF nanosheets, gauged at a thickness of 1.2 nm through meticulous morphological analysis (Figure 4b). These nanosheets displayed exemplary catalytic activity, positioning them as promising heterogeneous catalysts. Furthermore, the application of this technique extended to the creation of bimetallic CTGU‐10c_2_([NH_2_(CH_3_)_2_][Co_2_Ni_3_(μ_3_‐OH)(H_2_O)_3_(BHB)]) nanosheets.^[^ ^21^ ^]^ Manipulating the Co/Ni metal salt ratio yielded microcrystals with varying morphologies. A 1:2 ratio resulted in the thinnest nanosheets, measuring a mere 1.05 nm in thickness. Notably, these nanosheets exhibited superior electrocatalytic OER performance and sustained stability in an alkaline environment. The solvothermal synthesis method is simple to operate and prepares monolayer MOFs by reacting in organic solvents. It also allows control over characteristics such as morphology, crystal form, and pore structure by adjusting reaction conditions. However, problems with crystal aggregation and a lack of scalability in substrate selection might arise when organic solvents are used.

#### 3.2.3 Template Method

Template method utilizes presynthesized nanomaterials as templates. In the presence of the template, common synthesis methods (such as solvothermal) are employed to form the desired product. Finally, an appropriate method is selected to remove the template.^[^ ^99^ ^]^ Wang et al.^[^ ^100^ ^]^ used graphene oxide (GO) as a template and electronic medium to prepare ultrathin monolayer 2D MOF (Figure 4c). The synergistic interaction between the 2D MOF and GO electron conductor can significantly enhance the photocatalytic performance of CO_2_ reduction, with a CO selectivity of 95%, which is 34 times higher than that of bulk Co‐MOF. Additionally, the reduced graphene oxide can also serve as a 2D template support to stabilize the MOF. Template method can utilize predesigned templates as scaffolds to precisely control the size, shape, and structure of nanomaterials.^[^ ^101^ ^]^ Due to the ease of large‐scale preparation with templates of various sizes and uniform shapes, it is advantageous for the mass production of monolayer MOFs. However, after using the template method for material synthesis, it is necessary to choose appropriate removal methods, especially when there is a strong interaction between the template and the synthesized material, in order to avoid affecting the physical and chemical properties of the product.

#### 3.2.4 Surfactant‐Assisted Synthesis

Surfactant‐assisted synthesis is an effective bottom‐up approach for preparing monolayer 2D MOFs. Surfactants are organic compounds composed of polar hydrophobic groups and hydrophilic units, which can reduce the interfacial tension or surface tension between two phases, playing a crucial role in effectively controlling the size and shape of the target nanocrystals in MOFs.^[^ ^102^ ^]^ Surfactants (such as polyvinylpyrrolidone (PVP), cetyltrimethylammonium bromide (CTAB), and sodium dodecyl sulfate (SDS)) not only can restrict the growth of MOFs in the vertical direction, but also help stabilize the MOFs synthesized in solution, preventing the monolayer 2D MOF nanomaterials from reaggregating.^[^ ^103^, ^104^ ^]^ Qiu et al.^[^ ^105^ ^]^ used PVP as a surfactant to prepare copper(II)‐tetrakis(4‐carboxyphenyl)porphyrin (Cu‐TCPP) nanosheets through preassembly and coordination between Cu atoms (Figure 4d). The obtained ultrathin nanosheets have a more uniform size and higher yield compared to traditional exfoliation methods. Surfactant‐assisted synthesis can serve as a template or modifier during the growth process of MOFs, controlling the morphology, size, and structure of single‐layer MOFs. However, during the synthesis process, there may be some surfactant residues in the product that need to be further treated to remove residual substances. Currently, this method is mainly used for synthesizing few‐layer MOFs, with fewer reports on synthesizing monolayer 2D MOFs because preparing monolayer 2D MOFs requires more precise control and techniques.^[^ ^106^, ^107^ ^]^ In surfactant‐assisted synthesis, ensuring the formation of monolayer MOFs requires strict control over surfactant concentration, solution composition, temperature, and other factors to achieve stability and uniformity in the monolayer structure. With the increasing demand for applications of monolayer 2D MOFs in catalysis, sensing, and other fields, researchers’ studies on surfactant‐assisted synthesis of monolayer 2D MOFs will be further strengthened. It is believed that there will be more in‐depth research and reports in this field in the future.

### 3.3 Other Methods

Ligand exchange method refers to the process of combining MOF exfoliation and surface functionalization. Based on the principles of coordination chemistry, ligands with strong coordination ability replace those with weak coordination ability, resulting in the production of 2D monolayer MOF nanosheets from 3D MOFs. Deng et al.^[^ ^108^ ^]^ elucidated the fabrication of monolayer 2D MOF nanosheets possessing hydrophobic surfaces derived from layer‐supported 3D MOFs. This was achieved through the utilization of a high‐affinity coordination cap/ligand to supplant the low‐affinity coordination pillar ligand. Furthermore, leveraging the substantial concentration gradient of the former, they substituted the bpy pillar in two cadmium‐based multilayered pillar MOFs with alkylpyridine derivatives. The outcome was the development of monolayer 2D MOF nanosheets characterized by hydrophobic surfaces, exhibiting notable efficacy in oil and water separation. The ligand exchange method presents notable advantages, in terms of simplicity and cost‐effectiveness. However, challenges persist in the areas of ligand selection, controllability limitations, and the potential for structural alterations. Future investigations should prioritize the refinement of ligand exchange methodologies to elevate precision and controllability, thus mitigating the challenges inherent in practical applications.

## 4 The Applications of Monolayer 2D MOF Nanosheets

The applications of monolayer 2D MOF nanosheets are multifaceted, owing to their distinctive attributes encompassing a substantial surface area, abundant active sites, ordered porous structures, and an ultrathin single‐atom thickness. Consequently, they exhibit unique optical, electrical, and mechanical properties, making them highly promising for a wide range of applications. The tunable structure and functionality of monolayer 2D MOF nanosheets can be achieved by altering the metal nodes or organic ligands, providing advantages for specific applications. In recent years, significant progress has been made in fields such as catalysis, gas separation, chemical sensing, and energy storage with monolayer 2D MOFs. It's currently one of the most active and promising areas in research, with mature and rich research outcomes. These outcomes better demonstrate the performance and potential of monolayer MOF nanosheets. This section provides a brief summary of the applications associated with monolayer 2D MOF nanosheets.

### 4.1 Catalysis

#### 4.1.1 Photocatalysis

**Figure 5:**
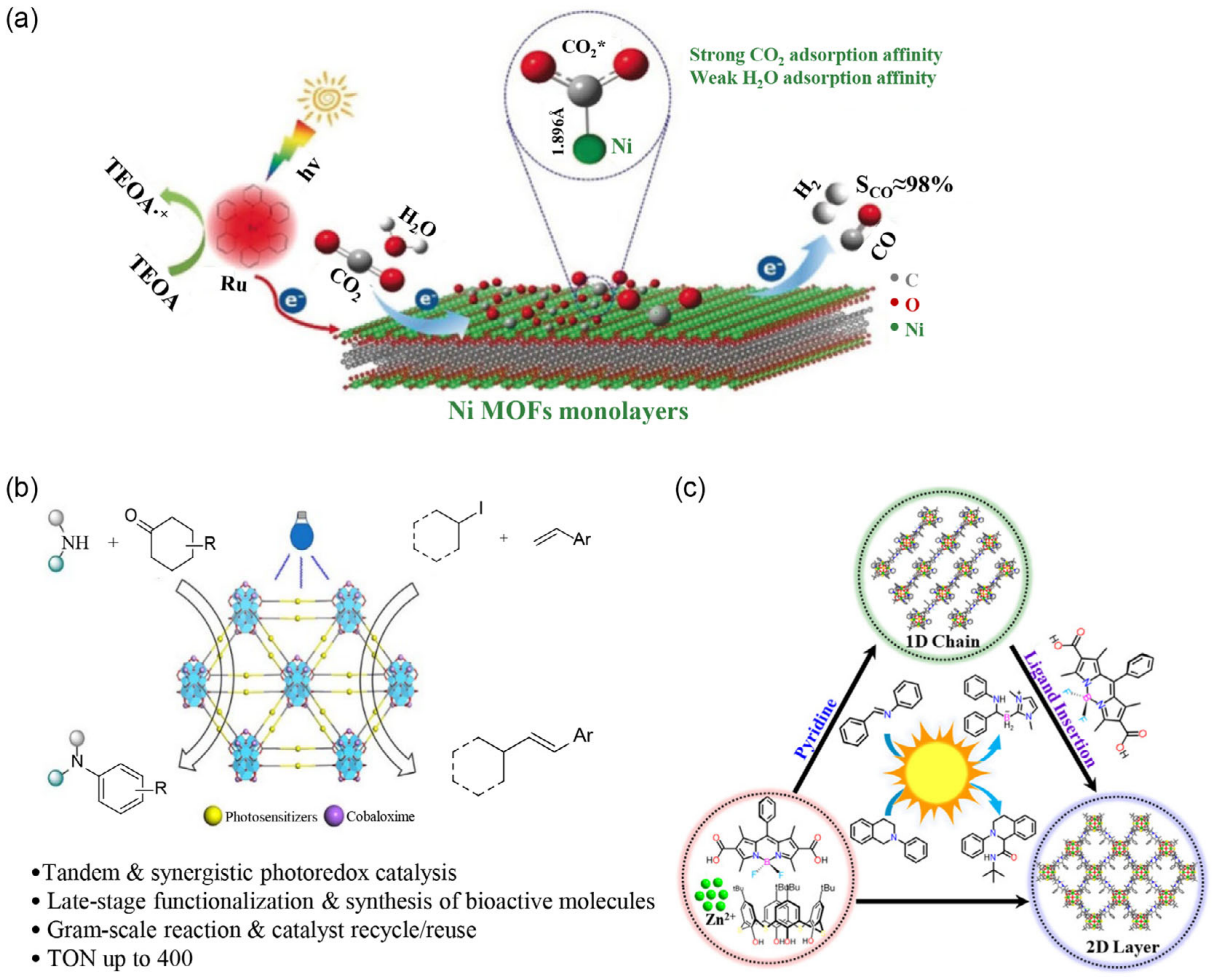
Applications of monolayer MOFs in photocatalysis. a) Schematic representation of monolayer nickel MOF nanosheets as a catalyst for CO_2_ photoreduction with dilution. Reproduced with permission.^[^ ^110^ ^]^ Copyright 2018, GDCh. b) Synergistic heck‐type coupling reaction catalyzed by multifunctional monolayer MOF containing photosensitizer through tandem dehydrogenation and coupling reactions. Reproduced with permission.^[^ ^112^ ^]^ Copyright 2023, ACS. c) Schematic illustration of photocatalytic diborane reduction and CDC reaction using monolayer 2D pyrrole–boron‐based MOF nanosheets. Reproduced with permission.^[^ ^113^ ^]^ Copyright 2023, ACS.

2D MOFs exhibit an ultrathin thickness and a high density of catalytic sites. In contrast to bulk MOFs, monolayer 2D MOFs possess exceptionally large specific surface areas and high‐density metal nodes. These features enhance light absorption, facilitating increased participation of photogenerated electrons in photocatalytic reactions, thereby expediting charge transfer. Consequently, monolayer 2D MOFs serve as an outstanding platform for designing multifunctional photocatalysts to meet diverse photocatalytic requirements. In recent years, the distinct properties of monolayer 2D MOFs have demonstrated expansive potential in the realm of photocatalysis.^[^ ^109^ ^]^ For instance, Han et al.^[^ ^110^ ^]^ showcased the utilization of monolayer 2D MOF nanosheets as photocatalysts for the photoreduction of diluted CO_2_ (**Figure** **5**a). Using an ultrasonication method, they prepared monolayer Ni MOF nanosheets with abundant coordinatively unsaturated Ni sites. The synthesized Ni MOFs comprised uniform ultrathin nanosheets with an average thickness of ≈1.0 nm. In diluted CO_2_ (10%) conditions, Ni MOFs exhibited the highest apparent quantum yield of 1.96% and a CO selectivity of 96.8%, surpassing previous reports in diluted CO_2_ systems. This study represents the first demonstration of metal node‐dependent performance in the photoreduction of diluted CO_2_. The specific structure and fully exposed active sites of monolayer 2D MOFs enable efficient mass transfer and maximize the utilization of active sites, showing great potential in flue gas photocatalytic reduction. Dong et al.^[^ ^111^ ^]^ designed monolayer Co‐MOF nanosheets with a thickness of less than 1 nm, which selectively convert CO_2_ into formic acid. The formic acid production efficiency of monolayer Co‐MOF (0.85 mmol g^−1^ h^−1^) is ≈13 times that of the bulk counterpart (0.065 mmol g^−1^ h^−1^), making it the highest reported in engineering for flue gas photoreduction. Additionally, monolayer 2D MOF nanosheets also exhibit significant potential in sustainable photocatalysis. They can be employed for sustainable tandem and synergistic photocatalytic oxidation–reduction reactions (Figure 5b).^[^ ^112^ ^]^ Furthermore, they demonstrate catalytic proficiency in efficient reverse‐borylation and cross‐dehydrogenative coupling (CDC) reactions (Figure 5c).^[^ ^113^ ^]^ The ability of monolayer 2D MOF nanosheets to achieve multifunctional catalysis by adjusting their structures and compositions, such as tandem and synergistic reactions, offers enhanced solutions for complex synthesis. Nevertheless, challenges persist in enhancing their stability, broadening the light absorption range, and managing production costs.

#### 4.1.2 Electrocatalysis

**Figure 6:**
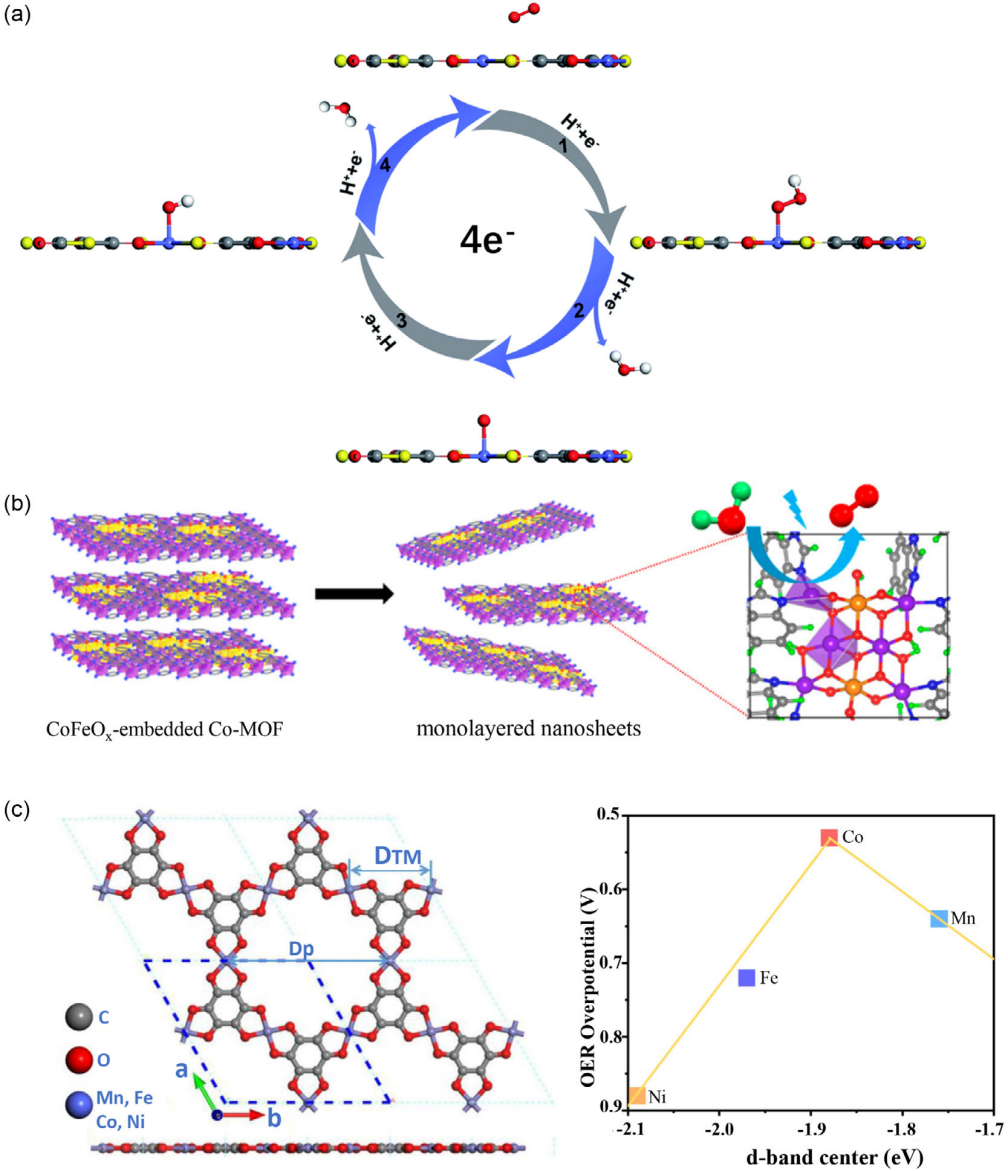
Applications of MOFs in electrocatalysis. a) Illustration of the oxygen reduction reaction (ORR) process occurring on the surface of monolayer 2D M_3_(C_6_S_3_O_3_)_2_ MOF. Reproduced with permission.^[^ ^119^ ^]^ Copyright 2021, RSC. b) Schematic representation of the synthesis and oxygen evolution reaction (OER) process of monolayer MOF nanosheets. Reproduced with permission.^[^ ^121^ ^]^ Copyright 2020, ACS. c) The views of TM‐O‐MOF monolayer structure and the volcano curve of OER overpotential as a function of the d‐band center. Reproduced with permission.^[^ ^122^ ^]^ Copyright 2022, MDPI.

Monolayer 2D MOF nanosheets seamlessly integrate the meticulously ordered structure of MOFs with the distinctive physicochemical attributes of 2D materials, rendering them highly versatile in the realm of electrocatalysis. The exceptionally tunable metal sites within monolayer 2D MOFs bestow the ability to regulate catalytic performance by varying the central metal, presenting a notable advantage in the development of promising electrocatalysts.^[^ ^114^, ^115^ ^]^ These materials find widespread application in critical electrocatalytic reactions, including the ORR, OER, and HER.^[^ ^116^, ^117^, ^118^ ^]^ For instance, Li et al.^[^ ^119^ ^]^ employed density functional theory calculations and molecular dynamics simulations to synthesize monolayer 2D MOFs of M_3_(C_6_S_3_O_3_)_2_ (M = Cr, Mn, Fe, Co, Ni, Cu, Pd, Rh, Ru) (**Figure** **6**a). The effective orbital coupling between the p‐conjugated organic linkers and the central metal renders all M_3_(C_6_S_3_O_3_)_2_ monolayers metallic. As catalysts for the ORR, M_3_(C_6_S_3_O_3_)_2_ monolayer 2D MOFs exhibit elevated limiting potentials and ORR activities. Rh_3_(C_6_S_3_O_3_)_2_, for instance, demonstrates a limiting potential of 0.81 V and an overpotential of 0.42 V, surpassing the performance of Pt/C. Different central metals exhibit distinct bond strengths with ORR intermediates and catalysts, enabling the modulation of catalytic performance through central metal variation. Additionally, Gao et al.^[^ ^120^ ^]^ presented findings on the ORR activity of Rh_3_(C_6_O_6_) monolayer 2D MOFs. The nanosheet exhibits an optimal adsorption strength for ORR intermediates, achieving a limiting potential of 0.80 V and demonstrating outstanding catalytic activity for the ORR. Furthermore, investigations into the application of monolayer 2D MOF nanosheets as OER catalysts have been documented. For example, Zhang et al.^[^ ^121^ ^]^ integrated ultrafine CoFeO_*x*_ nanoparticles with monolayer Co–N_4_‐based MOFs to fabricate OER electrocatalysts (Figure 6b). These inorganic nanoparticles were embedded into the Co‐based MOF lattice, subsequently exfoliated into layered heterostructures, resulting in monolayer heteronanosheets containing inorganic nanoparticles. Electrochemical investigations have revealed that ultrathin heterogeneous nanosheets, when deposited onto carbon cloth substrates, manifest outstanding electrocatalytic prowess in facilitating the OER at a current density of 10 mA cm^−2^. These nanosheets exhibit a remarkably low overpotential of 232 mV, concurrently achieving a peak current density of 55.2 mA cm^−2^ at 1.53 V. Notably, these nanosheets also showcase a modest Tafel slope of 32 mV dec^−1^. In contrast to the original MOF crystals, the synthesized OER electrocatalyst demonstrates heightened stability and substantially augmented activity, thereby expediting the sluggish oxygen evolution process. Previously reported performances of monolayer MOFs were mostly focused on specific electrocatalytic types. Li et al.^[^ ^122^ ^]^ delved into the examination of HER and OER performances within monolayer 2D transition metal oxide (TMO) MOFs, where TM represents Mn, Fe, Co, Ni. The catalytic efficacy of the nanosheets correlates with the d‐band of TM atoms, assigning the active sites for HER and OER to oxygen (O) and TM atoms, respectively (Figure 6c). Notably, the Co–O MOF demonstrates the highest catalytic activity, featuring a hydrogen atom adsorption Gibbs free energy (Δ*G* × *H*) of 0.02 eV and an OER overpotential of 0.53 V. Monolayer MOFs emerge as promising contenders in the realm of electrocatalysis. Future endeavors should concentrate on further enhancing the conductivity and electron transfer rate of MOF materials to attain more efficient electrocatalytic reactions. Moreover, addressing the stability concerns associated with MOF materials is imperative to ensure prolonged stability and recyclability.

### 4.2 Gas Separation

**Figure 7:**
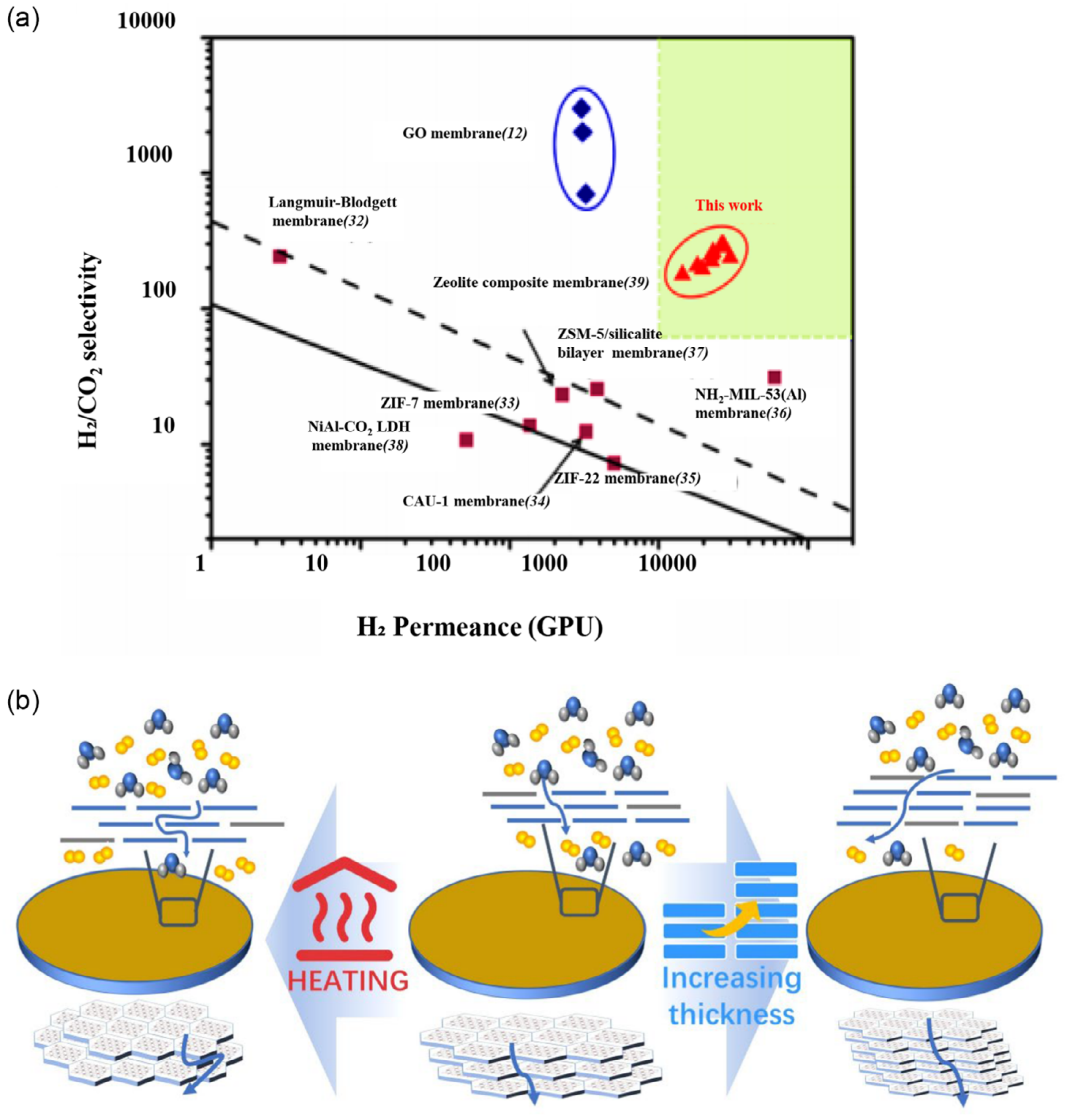
Applications of monolayer MOFs in gas separation. a) Performance evaluation of monolayer 2D Zn_2_(bim)_4_ nanosheets in H_2_ and CO_2_ separation. Reproduced with permission.^[^ ^70^ ^]^ Copyright 2014, AAAS. b) The 2D Zr‐BTB MOF nanosheets for tunable gas separation. Reproduced with permission.^[^ ^71^ ^]^ Copyright 2020, AAAS.

Gas separation technologies are of great significance for clean energy and sustainable development of the environment. Specifically, the storage of renewable energy sources such as hydrogen and natural gas requires the development of efficient adsorbent materials. One critical application is the purification of hydrogen in power plants, where the separation of H_2_ and CO_2_ constitutes a crucial step.^[^ ^123^, ^124^ ^]^ The remarkable properties of monolayer 2D MOFs, including high porosity, unique pore structure, open metal sites, and excellent permeability, establish a foundation for their utilization in gas separation and adsorption. Tailoring monolayer 2D MOF nanosheets to specific adsorbates enhances separation and adsorption efficiency.^[^ ^123^, ^124^ ^]^ For example, Peng et al.^[^ ^70^ ^]^ synthesized ultrathin molecular sieves using monolayer 2D MOF Zn_2_(bim)_4_ nanosheets as separation membranes for H_2_ and CO_2_. The nanosheets, characterized by a small pore size (0.21 nm), high crystallinity, and ultrathin features, demonstrated exceptional performance in H_2_ and CO_2_ separation (**Figure** **7**a). The membrane exhibited high selectivity for H_2_, boasting a hydrogen permeability of several thousand gas permeation units (GPUs) along with outstanding size selectivity. Notably, monolayer 2D Zn_2_(bim)_4_ nanosheets displayed superior thermal and hydrothermal stability, operating effectively under various temperature conditions for up to 400 h and enduring exposure to an equimolar H_2_/CO_2_ feed with ≈4 mol% steam at 150 °C for 120 h. Another example is the work of Li et al. Li and colleagues utilized a wet ball milling method to obtain monolayer Zr‐BTB nanosheets with a thickness of 1 nm.^[^ ^71^ ^]^ They fabricated ultrathin Zr‐BTB films on a GO porous carrier for H_2_/CO_2_ separation (Figure 7b). This membrane has adjustable separation performance, which can be achieved by adjusting the stacking amount of GO and MOF nanosheets perpendicular to the substrate and also by regulating the stacking mode of nanosheets in the horizontal direction through filtration under heating. At a film thickness of 130 nm, the selectivity for H_2_ over CO_2_ can be doubled, while the H_2_ permeability remains nearly unchanged (7.0 × 10^−7^ mol m^−2^ s^−1^ pa^−1^). Monolayer 2D MOF nanosheets have very broad prospects in future gas separation technologies due to their excellent separation performance, adjustable material properties, and potential environmental benefits.

### 4.3 Sensing Platforms

**Figure 8:**
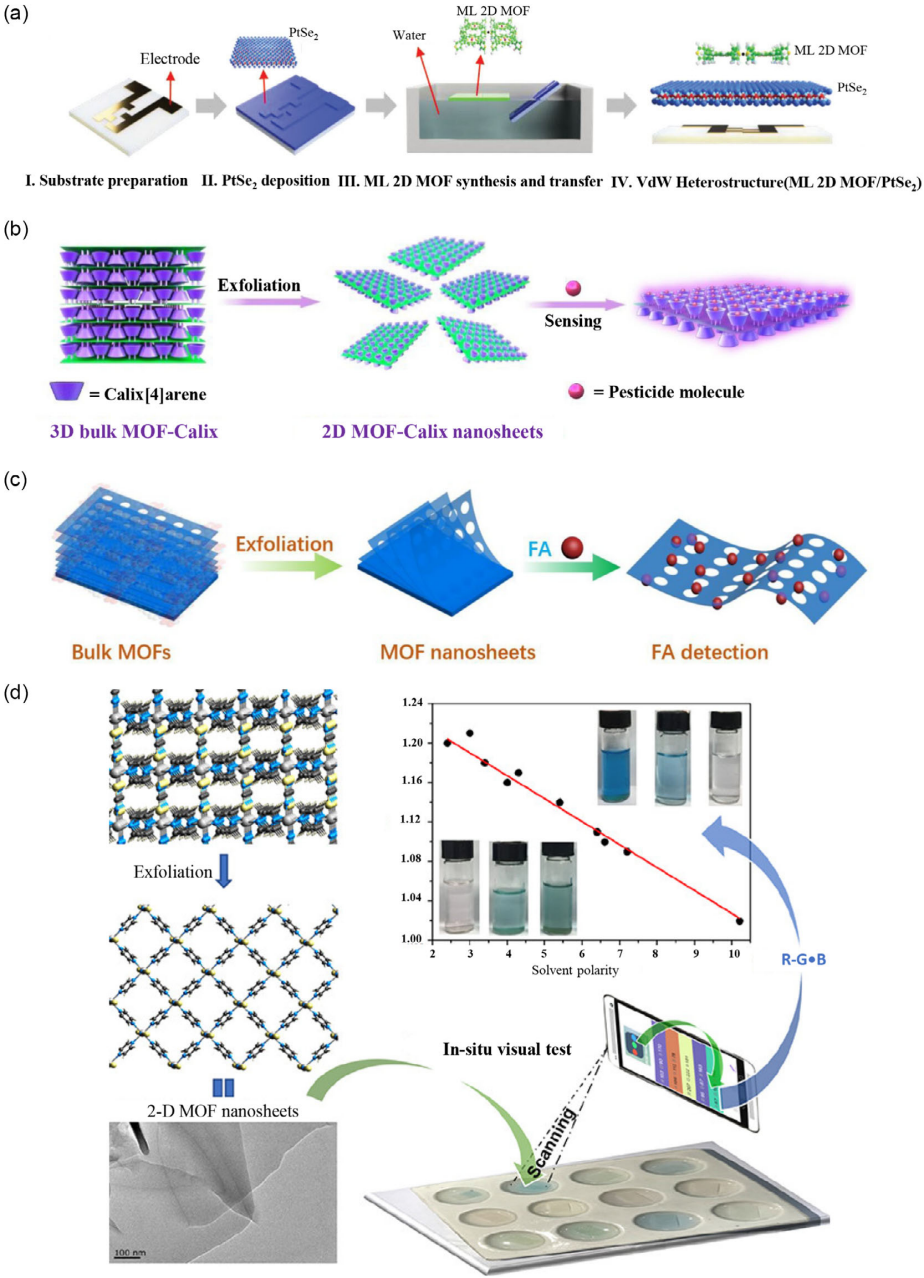
Applications of monolayer MOFs in sensing. a) Schematic representation of the fabrication process for 2D MOF‐PtSe_2_. Reproduced with permission.^[^ ^46^ ^]^ Copyright 2022, Wiley. b) Illustration of the preparation process for monolayer 2D MOF nanosheets and glyphosate detection. Reproduced with permission.^[^ ^129^ ^]^ Copyright 2020, Elsevier. c) Elucidation of the preparation process for monolayer 2D MOF nanosheets and folic acid detection. Reproduced with permission.^[^ ^50^ ^]^ Copyright 2023, Elsevier. d) Quantification of solvent polarity through detection using monolayer 2D MOF nanosheets.^[^ ^58^ ^]^ Copyright 2018, ACS.

Sensors represent indispensable instruments for acquiring information, playing pivotal roles across diverse sectors, including agricultural production, national defense, and various scientific and technological domains.^[^ ^24^, ^79^, ^125^, ^126^ ^]^ Recent advancements in nanomaterials have notably propelled the evolution of sensor functionalities, exemplified by enhanced stability, sensitivity, and selectivity.^[^ ^127^ ^]^ Monolayer 2D MOF nanosheets stand out with their ultrathin profiles, exceptional stability, outstanding electronic conductivity, and notable fluorescence quenching capabilities. These distinctive attributes render them highly versatile for applications in sensing platforms. Monolayer 2D MOF nanosheets find extensive utility across diverse sensing systems, encompassing gas sensors, fluorescence sensors, and nanopore biosensors.^[^ ^128^ ^]^ For instance, Cho et al.^[^ ^46^ ^]^ reported a breakthrough in gas selectivity within transition metal dichalcogenide (TMDs) gas sensors through the incorporation of monolayer 2D MOF nanosheets (**Figure** **8**a). The identifiable and precisely controllable geometric structure of these nanosheets, coupled with their atomic thickness, affords high‐quality gas permeability, facilitating selective filtration based on specific particle size permeation. Integration of monolayer 2D MOF nanosheets atop a PtSe_2_ gas sensor resulted in a shift of the primary sensing analyte from NO_2_ to H_2_S, while preserving the intrinsic ultralow detection limit of PtSe_2_. Notably, even at low gas concentrations (200 ppb), monolayer 2D MOFs sustain the heightened responsiveness of PtSe_2_, thereby significantly augmenting the gas selectivity of TMD gas sensors.

Furthermore, due to the prominently exposed active sites and the ultralarge surface area of monolayer 2D MOF nanosheets, they engage in direct surface interactions with analytes. Consequently, these nanosheets demonstrate exceptional fluorescence quenching capabilities, making them highly applicable in the realm of fluorescence sensors. Notably, Yu et al.^[^ ^129^ ^]^ engineered ultrathin monolayer 2D MOF nanosheets adorned with tetra‐pyridyl calix[4]arene, providing a selective and sensitive platform for glyphosate determination (Figure 8b). The tetra‐pyridyl calix[4]arene core encompasses a cavity, establishing an internal environment conducive to selective recognition, which can be affixed to the prepared 2D MOF nanosheets. The noncoordination of the pyridine group from the calix[4]arene ligand with Cd atoms permits free rotation via the methylene groups, thereby altering their size and shape. This self‐regulation of the cavity enhances binding with glyphosate molecules, augmenting the selectivity of glyphosate detection. The expansive surface area and readily accessible active sites on monolayer 2D MOF nanosheets facilitate efficient energy transfer from fluorescent molecules to quenchers, resulting in rapid detection and heightened sensitivity for glyphosate, with a detection limit as low as 2.25 μm. In a similar vein, Li et al.^[^ ^50^ ^]^ synthesized monolayer 2D MOF nanosheets endowed with distinctive fluorescent properties, leveraging them as sensors for detecting folic acid (Figure 8c). The exfoliation of 2D MOFs into monolayer nanosheets in ethanol through ultrasonication yielded enhanced fluorescence responsiveness. These nanosheets exhibited specific quenching toward folate molecules, showcasing a high quenching efficiency of 90% for folic acid and a detection limit of 0.112 μm.

In addition to the previously mentioned sensors, monolayer 2D MOF nanosheets offer a compelling avenue for the rapid and stable measurement of diverse polar solvents.^[^ ^130^, ^131^ ^]^ Notably, Luo et al.^[^ ^58^ ^]^ demonstrated the utility of monolayer 2D MOF nanosheets as an in situ visual medium for discerning solvent polarity (Figure 8d). The synthesized nanosheets exhibit excellent dispersibility across various solvents, resulting in stable colloidal suspensions and inducing distinctive color changes in the solvents. Specifically, in nonpolar solvents (e.g., benzene, toluene, and carbon tetrachloride), the colloidal suspension manifests a green hue. Conversely, in polar solvents (e.g., DMSO, acetone, and DMF), a vivid blue color characterizes the colloidal suspension. Each colloidal suspension displays unique absorption characteristics, wherein the *π* → π* transition band experiences a redshift corresponding to solvent polarity. Leveraging smartphones, monolayer 2D MOF nanosheets can serve as an in situ visual tool for quantitative and qualitative isomer identification, as well as determining the polarity of mixed solvents.

Monolayer MOFs hold immense promise in sensing applications, capable of detecting gases, ions, molecules, and various environmental substances. The development of high‐sensitivity and high‐selectivity monolayer MOF sensors is pivotal for achieving precise detection of trace targets, playing a pivotal role in shaping the future of sensor technology. Additionally, addressing challenges such as the stability of monolayer MOF sensors is imperative to enhance the practical feasibility of their applications.

### 4.4 Energy Storage

**Figure 9:**
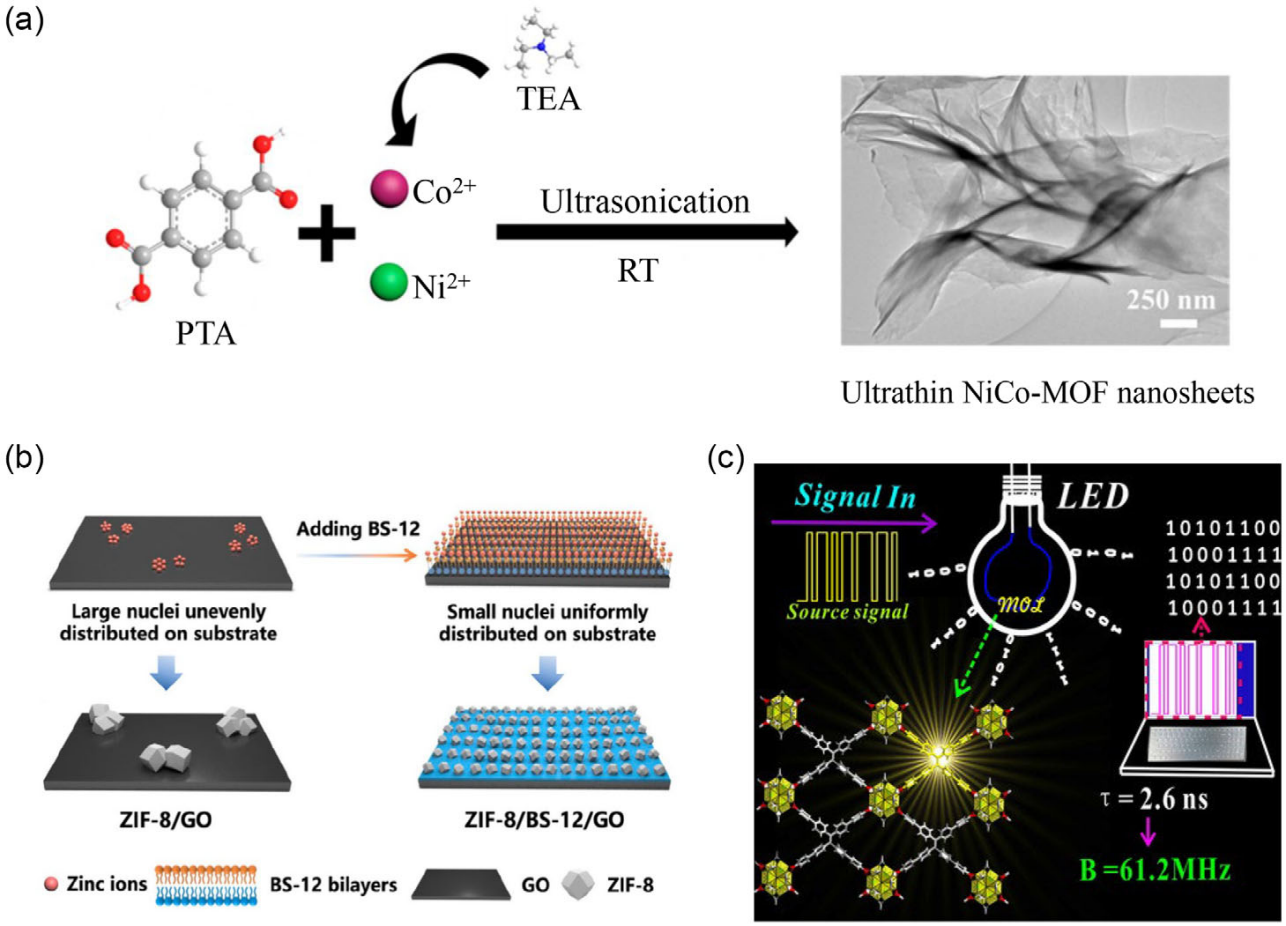
Applications of monolayer MOFs in energy storage. a) Preparation process of NiCo‐MOF. Reproduced with permission.^[^ ^136^ ^]^ Copyright 2019, ACS. b) Schematic illustration of the bidirectional electrostatic interaction‐induced coating of monolayer ZIF‐8 on GO sheets. Reproduced with permission.^[^ ^137^ ^]^ Copyright 2022, Wiley. c) 2D MOFs for fast white‐light communication. Reproduced with permission.^[^ ^139^ ^]^ Copyright 2017, Wiley.

Energy storage represents a pivotal domain in which monolayer 2D MOF nanosheets exhibit tremendous promise as advanced materials. Their intrinsic attributes, including a heightened surface area and porous structure, endow them with an expansive active surface area and efficient ion diffusion channels, culminating in outstanding capacitance performance. These materials find diverse applications across various domains such as supercapacitors, hydrogen storage, and integrated circuits,^[^ ^132^, ^133^, ^134^ ^]^ with supercapacitors standing out as the most extensive application. Monolayer MOFs are widely acknowledged as exemplary materials for constructing electrodes in supercapacitors due to their meticulously ordered porous structure and elevated specific surface area.^[^ ^135^ ^]^ Their superior electrochemical performance confers supercapacitors with augmented energy density and power density. For instance, in a study by Wang et al.^[^ ^136^ ^]^ Ni–Co–MOF nanosheets were meticulously prepared through ultrasound treatment and employed as electrode materials for supercapacitors. The resultant supercapacitor exhibited a specific capacitance of 1202.1 F g^−1^ at 1 A g^−1^, with a remarkable capacitance retention rate of 76.3% after 5000 cycles, attesting to its exceptional cycling stability (**Figure** **9**a). The ability of supercapacitors to maintain high energy storage efficiency and deliver stable electrical output over prolonged usage underscores the paramount importance of enhancing cycle stability.

In another study, Leng et al.^[^ ^137^ ^]^ precision coated monolayer MOFs on a series of substrates to fabricate MOF‐based nanocarbon composites for the cathode assembly of zinc‐ion hybrid capacitors. This approach resulted in a supercapacitor with a specific capacitance of 236 F g^−1^ at 0.5 A g^−1^ and a reaction rate of 98 F g^−1^ at 100 A g^−1^. Impressively, after 230 000 cycles, the capacitance retention rate reached 90.1%, exemplifying exceptional cycling stability (Figure 9b). The inherent orderliness of monolayer MOFs, coupled with the tunability of their porous structure, positions them as promising candidates for device fabrication, facilitating high integration and miniaturization. Notably, the application of monolayer MOFs extends to the realm of integrated circuits, where they contribute to the development of components such as white light‐emitting diodes. The device comprises p‐type and n‐type semiconductors, facilitating the direct conversion of electrical energy into light energy.^[^ ^138^ ^]^ As an illustrative instance, Hu et al.^[^ ^139^ ^]^ synthesized a monolayer 2D Zr‐TCBPE MOF. Upon integration into a chip to fabricate a white light emitting diode (WLED) device, the intrinsic modulation frequency of the chip was determined to be 1.7 MHz at −3 dB, exhibiting threefold enhancement compared to conventional WLEDs (Figure 9c). Additionally, the signal transmission rate of this WLED device surpassed that of commercial counterparts by a factor of 3. The pursuit of monolayer MOF materials exhibiting enhanced charge storage capacity is imperative for advancing the efficacy of energy storage and release. This endeavor holds paramount importance in extending the applicability of monolayer MOF nanosheets within the realm of energy storage.^[^ ^140^ ^]^ Nevertheless, the challenges of cyclic stability and durability inherent in monolayer MOF materials necessitate thorough consideration to realize enduring and sustainable energy storage devices.

### 4.5 Biomedicine

**Figure 10:**
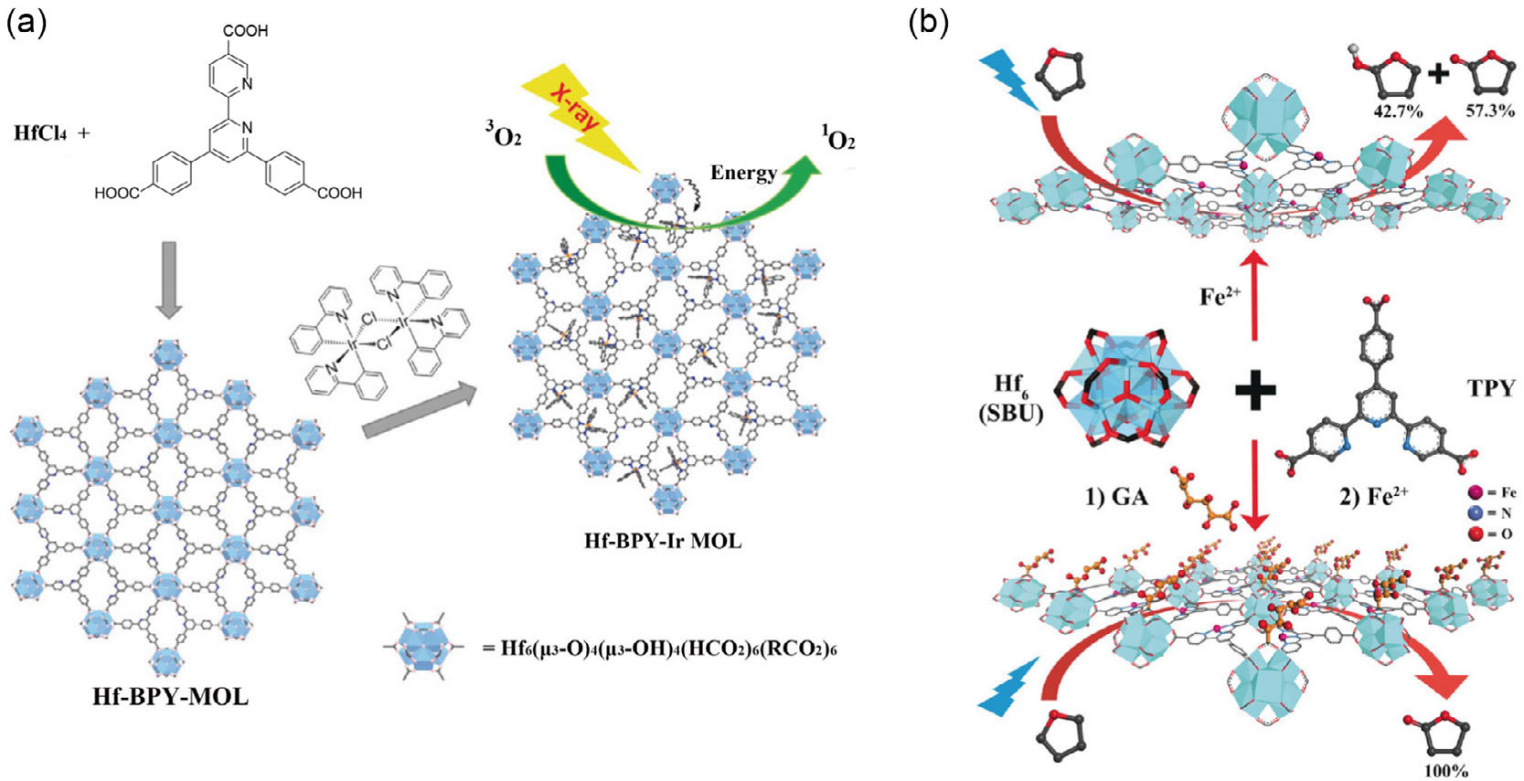
Applications of monolayer MOF nanosheets in the biomedical field. a) Schematic diagram of X‐PDT using MOL based on Hf and MOL for generating singlet oxygen. Reproduced with permission.^[^ ^142^ ^]^ Copyright 2017, Wiley. b) Simulation of the second catalytic center in iron‐containing monolayer 2D MOFs for aerobic oxidation of tetrahydrofuran, mimicking oxidases. Reproduced with permission.^[^ ^143^ ^]^ Copyright 2017, Wiley.

Conventional pharmaceuticals often exhibit limited targeting precision, resulting in suboptimal efficacy and the potential for severe side effects in clinical settings.^[^ ^141^ ^]^ Monolayer 2D MOF nanosheets, distinguished by their elevated specific surface area, offer a solution by enabling drug loading through mechanisms like electrostatic adsorption and *π*–*π* stacking. Characterized by facile functionalization and biodegradability, these nanosheets are rapidly advancing in the realm of biomedicine. Lan et al.^[^ ^142^ ^]^ recently detailed the utilization of monolayer MOF nanosheets in X‐ray‐induced photodynamic therapy (X‐PDT) for colon cancer (**Figure** **10**a). The nanosheets efficiently absorb X‐rays, initiating the generation of reactive oxygen species and enhancing PDT's efficacy. Experimental results underscored the potential of this treatment, showcasing a remarkable 90% reduction in tumor volume. This study underscores the promising application of monolayer 2D MOF nanosheets in biomedicine. Beyond cancer treatment, monolayer MOFs exhibit notable advantages in modulating enzyme microenvironments. For instance, Shi et al.^[^ ^143^ ^]^ employed monocarboxylate compound‐modified secondary building units of MOFs to engineer hydrophilic/hydrophobic microenvironments surrounding the reaction center, thereby creating a biomimetic catalytic milieu (Figure 10b). Monolayer MOFs present versatile applications in biomedicine, including drug delivery, cancer treatment, and tissue engineering. A prospective avenue for future research involves the development of monolayer MOF nanomaterials with enhanced drug loading capacities and controlled release properties, aiming for precise targeted therapy and drug delivery.

### 4.6 Other Applications

In addition to the aforementioned applications, monolayer 2D MOF nanosheets have also been used in the desalination of seawater. In today's global environment, ensuring clean and sustainable water resources is an urgent issue. The development of monolayer 2D MOF nanosheets can create energy‐efficient and environmentally friendly membranes for ion separation, which can be applied in the desalination process of seawater. For example, Jian et al.^[^ ^144^ ^]^ presented water‐stable monolayer 2D Al‐MOF nanosheets and showcased their efficacy as membranes for ion separation in water. Exfoliated Al‐MOF nanosheets exhibited prolonged structural integrity in aqueous environments and could be easily transformed into a laminar membrane through vacuum filtration on a porous substrate. This nanosheet displayed extremely low permeability to tested ions (≈3.3 × 10^−6^ mol m^−2^ h^−1^ bar^−1^), achieving nearly 100% rejection of inorganic ions at a high water flux of 2.2 mol m^−2^ h^−1^ bar^−1^. Furthermore, due to the parallel *π*–*π* interactions that self‐lock the interlayer distance in Al‐MOF layered membranes, they can operate stably for over 750 h, offering possibilities for enhancing the selectivity of desalination membranes. Solar cells, as a clean and renewable energy technology, have significant importance. In comparison to traditional silicon‐based solar cells, organic solar cells have unique advantages, including lower manufacturing costs, large‐scale production capabilities, and the ability to create flexible, stretchable, and transparent devices, representing a new direction in photovoltaic technology development.^[^ ^145^ ^]^ Foster et al.^[^ ^146^ ^]^ were the first to achieve the scalable synthesis of monolayer Zn_2_(H_2_TCPP) (H_2_TCPP = meso‐tetra(4‐carboxyphenyl)porphine) with a yield of up to 99% using a continuous stirred‐tank reactor, which was subsequently utilized in organic solar cells. The synthesized monolayer MOF nanosheets were incorporated into the active layer of organic photovoltaic cells, resulting in a doubling of the device's power conversion efficiency. This study provides a low‐cost, high‐performance composite material for organic solar cell devices, opening up new possibilities for the application of organic solar cells.

## 5 Conclusions and Perspectives

In conclusion, this manuscript offers a comprehensive review of diverse fabrication methodologies for monolayer 2D MOF nanosheets, highlighting their recent applications in gas separation, catalysis, sensing platforms, and energy storage. In recent years, various methods for synthesizing monolayer 2D MOFs have been explored and developed, providing a broader spectrum of choices and possibilities for the fabrication of monolayer 2D MOFs. Through the utilization of distinct principles and techniques, the synthesis of monolayer 2D MOFs has been successfully achieved, demonstrating substantial potential for enhancing synthesis efficiency while providing control over structures and properties. This establishes a robust foundation for their applications in catalysis, gas separation, sensing, and other domains of scientific inquiry. At present, techniques such as interface‐assisted synthesis methods have emerged as promising avenues for the synthesis of monolayer MOFs. These approaches offer improved preservation of the MOF structure and enable precise control over size and thickness. Nonetheless, the choice of methodology hinges on specific application requirements and material properties, each method presenting distinct scopes of applicability and associated advantages and disadvantages. Therefore, careful consideration must be given to selecting the most suitable method tailored to specific circumstances and needs. Looking forward, from an industrial production standpoint, the future trajectory likely favors methodologies that are efficient, controllable, scalable, and cost‐effective. These methodologies should possess the production capacity and stability requisite to meet industrial demands, thereby enabling large‐scale manufacturing of high‐quality monolayer MOF products. On the other hand, from a fundamental research perspective, future mainstream methodologies are anticipated to offer flexibility, diversity, and tunability in synthesis. Such methodologies should cater to researchers’ requirements for precise control over the properties and structures of monolayer MOF materials, advancing our comprehension of MOF materials and facilitating their broader applications.

Despite the advancement of fabrication techniques, challenges and limitations persist in meeting the demanding requirements for high‐efficiency production of monolayer 2D MOF nanosheets. The identified drawbacks are delineated as follows: 1) Achieving precise control over the intricate structure of monolayer 2D MOFs necessitates meticulous experimental conditions and presenting challenges in their synthesis and manipulation; 2) Top‐down methods for nanosheet extraction exhibit low efficiency and stability, as the intralayer coordination bonds of layered 2D MOF nanosheets may not always be stronger than interlayer interactions, impeding the exfoliation of certain 2D MOF nanosheets. The most unstable sites in MOFs are the coordination bonds linking organic ligands and metal clusters. The instability of these coordination bonds poses significant challenges in their preparation and characterization. Additionally, during the exfoliation process, MOFs may tend to stack, potentially compromising their performance and applications; and 3) Bottom‐up methods can control the morphology of the product by adjusting the reaction time. However, it is difficult to separate surfactant molecules or end ligands from the surface of nanosheets, and in most cases, the yield is low. Despite the broad application potential of monolayer 2D MOF nanosheets across diverse fields, efficient fabrication of high‐quality monolayer 2D MOF nanosheets remains a prevalent technical challenge. Future research endeavors will prioritize the development of more efficient synthetic methods and processes to achieve controlled and scalable production of monolayer 2D MOF nanosheets. This entails the design and synthesis of innovative ligands and metal ions, optimization of reaction conditions, and feasibility studies for industrial‐scale production.

Monolayer 2D MOF nanosheets have demonstrated remarkable achievements across diverse application domains; nevertheless, comprehensive exploration of their applications is still nascent. The stability of monolayer 2D MOFs is a key prerequisite for their practical applications in catalysis, gas separation, sensing, biomedicine, and other fields. This stability mainly includes chemical, thermal, and mechanical stability. The chemical stability of MOFs ensures that they remain intact and functional when exposed to various chemical environments, preventing degradation or structural changes.^[^ ^147^ ^]^ Thermal stability and mechanical stability allow MOFs to maintain structural integrity when exposed to high temperatures, vacuum, or pressure.^[^ ^148^ ^]^ However, most reported monolayer 2D MOFs exhibit low durability in the aforementioned operating environments, greatly hindering their practical applications. To enhance the overall stability of monolayer 2D MOFs, several strategies can be used. These include selecting building blocks with strong coordination bonds, optimizing synthesis methods to reduce defects, and increasing surface hydrophobicity to prevent water adsorption into pores or condensation around metal clusters, thereby enhancing the water stability of monolayer 2D MOFs. By addressing these stability issues, monolayer 2D MOFs can further unleash their potential in various applications. Future endeavors in the extensive investigation of monolayer 2D MOF nanosheets are anticipated to reveal more profound and comprehensive developmental trends. Their distinct advantages are evident in the following aspects: 1) Larger external surface area and more exposed active sites: Monolayer 2D MOF nanosheets, with their larger external surface area and increased exposed active sites compared to multilayer structures, exhibit heightened activity in adsorption, catalysis, and other surface reactions. This property finds applications in CO_2_ capture, natural gas purification, and addressing challenges related to environmental and energy concerns; 2) Tunable structure and function: Due to the tunable porous structure and functional groups capable of coordinating with metal ions in monolayer 2D MOF nanosheets, it is possible to achieve a high level of structural customizability by selecting suitable bridging ligands and metal ions according to different applications; 3) Optical properties for device fabrication: Leveraging their unique structure and outstanding optical properties, monolayer 2D MOF nanosheets can be employed in crafting high‐performance electronic devices such as photovoltaic converters, sensors, and supercapacitors. By preparing a monolayer structure, it is possible to achieve more precise control and adjustment of the material properties, thereby meeting the needs of different application fields; 4) High flexibility and expandability: Monolayer 2D MOF nanosheets exhibit good flexibility and expandability, making them valuable in flexible electronic devices, catalyst supports, separation membranes, and other fields. Additionally, the monolayer structure can be combined with other materials to further expand its range of applications; and 5) Biocompatibility for biomedical applications: With commendable biocompatibility and adjustable pore structures, monolayer 2D MOF nanosheets find suitability in biomedical applications, including drug delivery, bioimaging, and cell repair.

In conclusion, while monolayer 2D MOFs encounter challenges in their early developmental stages, ongoing research and exploration are poised to surmount these obstacles. The anticipated rapid progression of monolayer 2D MOF research holds promise for uncovering its substantial potential as an innovative 2D material.

## Conflict of Interest

The authors declare no conflict of interest.
